# *Xanthomonas campestris* FabH is required for branched-chain fatty acid and DSF-family quorum sensing signal biosynthesis

**DOI:** 10.1038/srep32811

**Published:** 2016-09-06

**Authors:** Yong-Hong Yu, Zhe Hu, Hui-Juan Dong, Jin-Cheng Ma, Hai-Hong Wang

**Affiliations:** 1 Guangdong Provincial Key Laboratory of Protein Function and Regulation in Agricultural Organisms, College of Life Sciences, South China Agricultural University, Guangzhou, Guangdong 510642, China; 2 Guangdong Food and Drug Vocational College, Guangzhou, Guangdong 510520, China

## Abstract

*Xanthomonas campestris* pv. *campestris* (*Xcc*), a Gram-negative phytopathogenic bacterium, causes black rot disease of cruciferous vegetables. Although *Xcc* has a complex fatty acid profile comprised of straight-chain fatty acids and branched-chain fatty acids (BCFAs), and encodes a complete set of genes required for fatty acid synthesis, there is still little known about the mechanism of BCFA synthesis. We reported that expression of *Xcc fabH* restores the growth of *Ralstonia solanacearum fabH* mutant, and this allows the *R. solanacearum fabH* mutant to produce BCFAs. Using *in vitro* assays, we demonstrated that *Xcc* FabH is able to condense branched-chain acyl-CoAs with malonyl-ACP to initiate BCFA synthesis. Moreover, although the *fabH* gene is essential for growth of *Xcc*, it can be replaced with *Escherichia coli fabH*, and *Xcc* mutants failed to produce BCFAs. These results suggest that *Xcc* does not have an obligatory requirement for BCFAs. Furthermore, *Xcc* mutants lost the ability to produce *cis*-11-methyl-2-dodecenoic acid, a diffusible signal factor (DSF) required for quorum sensing of *Xcc*, which confirms that the fatty acid synthetic pathway supplies the intermediates for DSF signal biosynthesis. Our study also showed that replacing *Xcc fabH* with *E. coli fabH* affected *Xcc* pathogenesis in host plants.

Fatty acid synthesis (FAS) is a vital metabolic pathway in all organisms, except the *Archaea*^1^,^2^. In most bacteria, fatty acid synthase (FAS II) is composed of a series of discrete, small, soluble proteins, and each enzyme, which is encoded by a separate gene, catalyzes a single step in the biosynthetic pathway^1^,^2^,^3^,^4^. The FAS II pathway of bacteria not only produces a diversity of products, including saturated fatty acids, unsaturated fatty acids, branched chain fatty acids (BCFAs), and hydroxylated fatty acids, for cellular structures^1^,^2^, but also supplies intermediates used in the synthesis of other end products, such as the cofactors lipoate^5^, biotin^6^,^7^, and quorum sensing signal molecules^8^,^9^,^10^,^11^.

The FAS II pathway in *Escherichia coli* has been extensively investigated, and it provides an almost complete description of the mechanisms that govern the synthesis of fatty acids^1^,^2^,^3^,^4^. Using similar pathways^1^,^2^,^12^, many Gram-positive organisms, such as the bacillus, staphylococci, and streptomycetes, are able to produce BCFAs with odd- and even-numbered carbon chains^13^,^14^,^15^,^16^. However, the substrate specificity of 3-ketoacyl-acyl carrier protein (ACP) synthase III (FabH) in Gram-positive bacteria is distinct from that in *E. coli*. The *E. coli* FabH has a strong preference for acetyl-CoA as a primer, which leads to the only production of straight-chain fatty acids in this organism^2^. In *Listeria monocytogenes*, *Bacillus subtilis*, and many Gram-positive bacteria, the FabH proteins are highly selective in accepting isovaleryl-CoA, isobutyryl-CoA, and 2-methylbutyryl-CoA as primers, and primarily iso- and anteiso-BCFAs are produced in these bacteria^17^,^18^,^19^ (Fig. 1A).

Generally, BCFAs are common components of Gram-positive bacteria that are often absent in Gram-negative bacteria^12^,^20^. However, at least 10 bacterial genera, including *Xanthomonas*, *Legionella*, *Flavobacterium*, *Bacteroides*, and *Desulfovibrio*, of Gram-negative bacteria have been reported to produce BCFAs^20^. The *Xanthomonas* genus is one of the most ubiquitous groups of plant-associated bacterial pathogens, which have been shown to infect at least 124 monocotyledonous and 268 dicotyledonous plant species, many of which are economically important crops or plants^21^. *Xanthomonas* has a complex fatty acid profile comprised of straight-chain saturated fatty acids, unsaturated fatty acids, and BCFAs^22^,^23^,^24^. Although the fatty acid profiles of *Xanthomonas* have been investigated for taxonomic purposes^22^,^23^,^24^, and several fatty acid synthetic enzymes, including FabD, FabB, FabH, and FabV from *Xanthomonas oryzae* pv. *oryzae*^25^,^26^,^27^,^28^, have been expressed and crystallized, little is known about the fatty acid biosynthetic pathway in *Xanthomonas*.

*Xanthomonas campestris* pv. *campestris* (*Xcc*) is the causal agent of black rot disease, one of the most destructive diseases of cruciferous vegetables worldwide^21^,^29^. This bacterium uses quorum sensing (QS) mechanisms mediated by molecules of the diffusible signal factor (DSF) family to regulate the expression of factors that contribute to virulence^8^,^21^. The DSF signal family is a novel structural class of QS signals with the *cis*-2-unsaturated fatty acid moiety, which includes *cis*-11-methyl-2-dodecenoic acid (11-Me-C_12_:Δ^2^), *cis*-2-dodecenoic acid (C_12_:Δ^2^), *cis*-11-methyldodeca-2,5-dienoic acid (11-Me-C_12_: Δ^2,5^), and *cis*-10-methyl-2-dodecenoic acid (10-Me-C_12_:Δ^2^) in *Xcc*^8^,^30^,^31^ (Fig. 1B). Genetic analyses show that *Xcc rpfF* encodes a key enzyme for biosynthesis of the DSF family signals^32^. Furthermore, using *in vitro* assays, RpfF is shown to be a bifunctional enzyme that catalyzes not only dehydration of 3-hydroxydodecanoyl-ACP to *cis*-2-dodecenoyl-ACP, but also cleaves the thioester bond to produce C_12_:Δ^2^, a DSF family member^8^,^9^ (Fig. 1B). This suggests that the DSF family signals synthetic precursors come from the fatty acid biosynthetic pathway^8^,^9^. According to this hypothesis, to produce 11-Me-C_12_:Δ^2^, another main DSF family signal, *Xcc* should synthesize 11-methyl-3-hydroxydodecanoyl-ACP first, which is an intermediate of the BCFA synthetic pathway (Fig. 1B). However, although *Xcc* encodes a complete set of genes required for fatty acid synthesis^33^, there is still little known about the mechanism of BCFA synthesis, especially the substrate specificity of *Xcc* FabH.

In the present study, we use several molecular biological techniques, including genetic complementation, biochemical analysis, and gene knockout, to characterize the functions of *Xcc* FabH in BCFA synthesis and DSF family signal production in *Xcc*. We found that FabH is a key BCFA biosynthetic enzyme in *Xcc*. Moreover, we confirm that *Xcc* FabH, like FabH in many Gram-positive bacteria, also prefer branched-chain acyl-CoAs as primers to initiate fatty acid synthesis. We also report that replacement of *Xcc fabH* with *E. coli fabH* results in the loss of the ability of *Xcc* to produce 11-Me-C_12_:Δ^2^, and this further provides *in vivo* evidence that the precursors of DSF family signals shunt from the fatty acid biosynthetic pathway.

## Results

### Bioinformatics analysis of *Xcc* FabH

**Figure 1:**
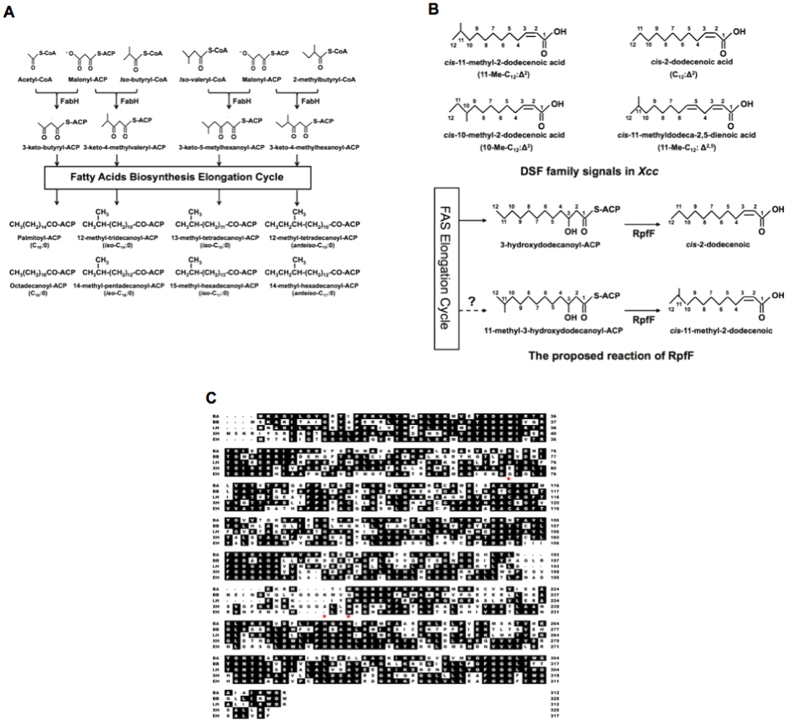
Initiation of bacterial fatty acid biosynthetic pathway with various acyl-CoA precursors, the proposed reaction of RpfF and alignment of *Xcc* FabH with FabHs from other bacteria. (**A**) Acetyl-coA, isobutyryl-CoA, isovaleryl-CoA, or 2-methylbutyryl-CoA utilized by FabH as starting units to initiate fatty acid biosynthesis. FabH indicates 3-ketoacyl-ACP synthase III. (**B**) DSF family signals in *Xcc* and the proposed reaction of RpfF. (**C**) Alignment of *Xcc* FabH with FabHs from other bacteria. The alignment was done with Clustal W, based on identical amino acid residues. BA, *B. subtilis* FabH1; BB, *B. subtilis* FabH2; XH, *Xcc* FabH; EH, *E.coli* FabH. The active sites of FabH, Cys-His-Asn, are marked with asterisks.

To investigate the *Xcc* FabH function in BCFA synthesis, alignments of *Xcc* FabH with FabH proteins from *E. coli*^34^, *L. monocytogenes*^17^, and *B. subtilis*^19^ were examined (Fig. 1C). The results showed that the Cys-His-Asn catalytic triad of 3-ketoacyl-ACP synthase III^2^ is conserved in *Xcc* FabH, and the *Xcc* FabH protein shares 61.2%, 47.2%, 45.3%, and 41.5% identical residues with *E. coli* FabH, *L. monocytogenes* FabH, *B. subtilis* FabHA, and *B. subtilis* FabHB, respectively (Fig. 1C). We also aligned *Xcc* FabH with *Ralstonia solanacearum* FabH^35^, and *Streptomyces coelicolor* FabH^16^, and the respective identity values were 56.1% and 36.3% (data not shown). Interestingly, both *E. coli* and *R. solanacearum* produce only straight-chain fatty acids^12^,^36^, while *L. monocytogenes*, *B. subtilis*, and *S. coelicolor* are able to synthesize several BCFAs^18^,^19^,^37^. Because the *Xcc* FabH protein has higher amino acid identities when compared to the straight-chain fatty acid producing bacterial FabHs, than to the BCFA producing bacterial FabHs, it would be expected that *Xcc* FabH would prefer acetyl-CoA as a primer to initiate straight-chain fatty acid synthesis, rather than it playing a role in BCFA synthesis.

### The *Xcc fabH* gene complements the *R. solanacearum fabH* deletion mutant

**Figure 2:**
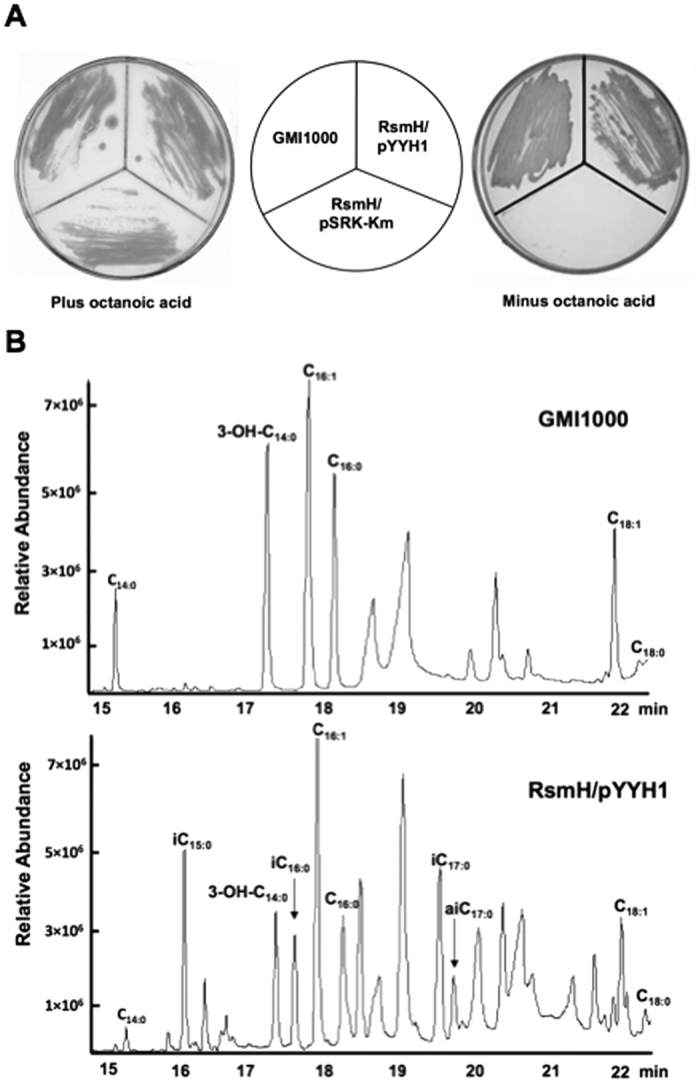
Expression of *Xcc fabH* restores growth of *R. solanacearum fabH* knockout strain RsmH and fatty acid profiles of strain RsmH/pYYH1. (**A**) *R. solanacearum fabH* mutant strain RsmH harboring pYYH1 that encoded *Xcc fabH* was grown on BG medium in the absence of octanoic acid. (**B**) Fatty acid profiles of RsmH/pYYH1. iC_15:0_, 13-methyl-tetradecanoic acid; 3-OH-C_14:0_, 3-hydroxyl-tetradecanoic acid; C_16:1_, palmitoleic acid; C_16:0_, palmitic acid; iC_17:0_, 15-methyl-palmitic acid; aiC_17:0_, 14-methyl-palmitic acid; C_18:1_, *cis*-11-octadecenoic acid.

To confirm the above hypothesis, the plasmid pYYH1, in which the *Xcc fabH* was expressed from the *E. coli lac* promoter in vector pSRK-Km^38^, was introduced into the *R. solanacearum fabH* mutant strain RsmH by conjugation. The *R. solanacearum* RsmH strain is an octanoic acid auxotrophic mutant, in which the *R. solanacearum* genomic *fabH* gene contains an in-frame deletion^35^. This strain does not grow in medium in the absence of octanoic acid (Fig. 2A). The derivative of RsmH containing the plasmid pYYH1 grew well on BG medium in the absence of octanoic acid, whereas the strain RsmH derivative carrying the vector plasmid failed to grow under the same conditions (Fig. 2A). This indicated that *Xcc fabH* encodes a 3-ketoacyl-ACP synthase III functional in *R. solanacearum*. To investigate the function of the *Xcc* FabH in fatty acid synthesis, the fatty acid composition of RsmH containing the pYYH1 plasmid was determined by gas chromatography-mass spectrometry (GC-MS). *R. solanacearum* GMI1000 does not produce BCFAs^36^ (Fig. 2B). However, the fatty acid profile of RsmH containing pYYH1 differed from the *R. solanacearum* wild type GMI1000 strain, and contained more than 33.8% BCFAs, which mainly included *iso*-C15, *iso*-C16, *iso*-C17, and *anteiso*-C17 fatty acids (Fig. 2B and Table S3). This suggested that *Xcc* FabH could use branched-chain acyl-CoAs, such as isovaleryl-CoA, isobutyryl-CoA, or 2-methylbutyryl-CoA, as substrates to produce BCFAs *in vivo*.

### *Xcc* FabH exhibits a wide range of substrate specificities *in vitro*

**Figure 3:**
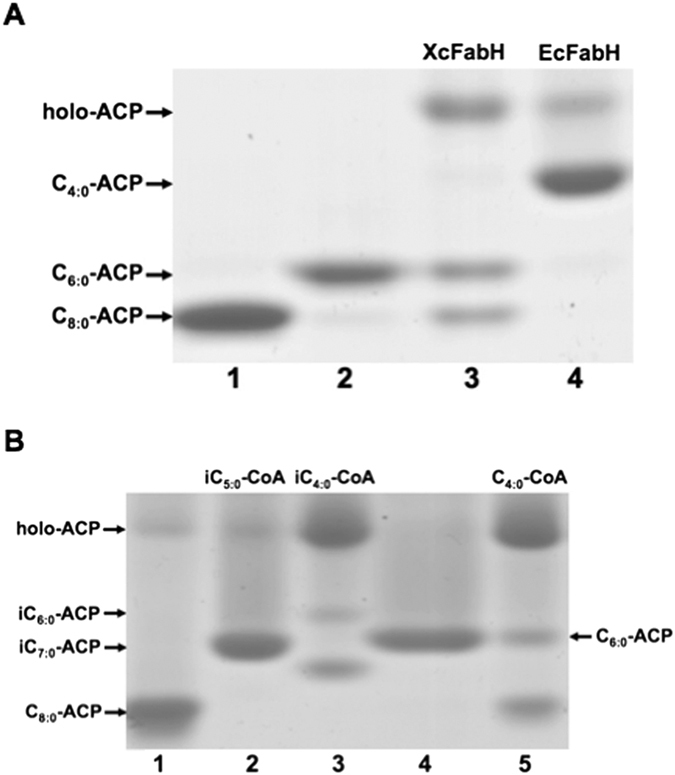
Enzymatic characterization of *Xcc* FabH in fatty acid biosynthesis *in vitro*. (**A**) The initiation fatty acid synthesis was reconstructed using a combination of *E. coli* FabZ, FabG, FabI, and *Xcc* FabH (lane 3) or *E. coli* FabH (lane 4) with NADH, and NADPH as cofactors, and malonyl-ACP plus acetyl-CoA as substrates. The migration positions of hexanoyl-ACP (C_6:0_-ACP, lane 2) and octanoyl-ACP (C_8:0_-ACP, lane 1) on gel are shown. (**B**) The initial reaction of fatty acid synthesis contained *E. coli* FabZ, FabG, FabI, and Xcc FabH, NADH, and NADPH as cofactors, and malonyl-ACP plus isobutyryl-CoA (lane 3), isovaleryl-CoA (lane 2) or butyryl-CoA (lane 5) as substrates.

In order to perform a direct *in vitro* assay of *Xcc* FabH substrate specificity, the initial reaction was reconstituted by addition of *E. coli* fatty acid synthetic enzymes (including FabD, FabG, FabZ, and FabI), *Xcc* FabH, and an acyl-CoA as described in “Materials and Methods”. First, we tested *Xcc* FabH using acetyl-CoA as a primer to initiate fatty acid synthesis (Fig. 3A). As expected the reaction containing *E. coli* FabH produced butyryl-ACP (Fig. 3A, lane 4). However, although the *Xcc* FabH reaction resulted in the appearance of new bands on conformationally sensitive gels, butyryl-ACP was not a product. Surprisingly, *Xcc* FabH converted acetyl-CoA to hexanoyl-ACP and octanoyl-ACP (Fig. 3A, lane 3). This suggested that *Xcc* FabH not only uses acetyl-CoA as its primer to initiate fatty acid synthesis, but also condenses short-chain acyl-ACPs with malonyl-ACP to produce longer acyl-ACPs.

*Xcc* FabH also used branched-chain acyl-CoAs, isobutyryl-CoA, or isovaleryl-CoA, as a primer to initiate fatty acid synthesis (Fig. 3B, lanes 2 and 3). We also tested if *Xcc* FabH utilized butyryl-CoA, hexanoyl-CoA, octanoyl-CoA, or decanoyl-CoA as a primer to initiate fatty acid synthesis, and found that each of these acyl-CoAs could be converted into longer acyl-ACPs by *Xcc* FabH (Figs 3B and S1). Hence, *Xcc* FabH can use a wide range of acyl-CoAs as the primer substrate. In order to probe the substrate specificity of *Xcc* FabH, the decrease in absorbance of NADPH at 340 nm was monitored spectrophotometrically in reaction mixtures containing holo-ACP, malonyl-CoA, NADPH, *E. coli* FabD and FabG, *Xcc* FabH, and various acyl-CoAs (C_2_–C_12_) (Table 1). *Xcc* FabH exhibits a low activity with acetyl-CoA but a high activity with isobutyryl-CoA or isovaleryl-CoA, consistent with the FabH enzyme activities of bacteria that produce BCFAs^17^,^19^ (Table 1). However, *Xcc* FabH had higher activities with 4- to 8-carbon straight chain acyl-CoAs than with branched-chain acyl-CoAs (Table 1). The best *Xcc* FabH substrate was butyryl-CoA. As the chain length of the acyl-CoA increased, the activities of *Xcc* FabH decreased. These results showed that *Xcc* FabH has a slight preference for short, medium-straight chain acyl-CoAs as a primer to initiate fatty acid synthesis and has the ability to use various acyl-CoAs (including short, medium-straight chain, and branched-chain acyl-CoAs) as primers. This characteristic distinguishes the *Xcc* FabH enzyme from *E. coli* and *B. subtilis* FabH enzymes^17^,^19^,^34^.

### *Xcc* requires *fabH* for growth

In order to identify the physiological function of *Xcc fabH* in BCFA synthesis, we attempted to disrupt the *Xcc fabH* gene in the *Xcc* genome. First, we constructed an in-frame deletion suicide plasmid, pZTT-1, according to the strategy described in Fig. S2A. After the plasmid pZTT-1 was introduced into the *Xcc* wild type strain 8004, *Xcc fabH* deletion mutants were selected according the methods described in the “Material and Methods”. Although several independent experiments were done, no *Xcc fabH* deletion strain was isolated. Only the single crossover integrant, *Xcc* YH1, was isolated (Fig. S2B,C), suggesting that *Xcc fabH* is an essential gene for *Xcc* growth.

To confirm this possibility, a new suicide plasmid pYYH-3 was constructed, in which an inner 550 bp DNA fragment of *Xcc fabH* was inserted into the plasmid pK18mobsacB (Fig. S3). The plasmid pYYH-3 was introduced into *Xcc* wild type strain 8004 by conjugation, and the conjugants were selected on nutrient glycerol medium (NYG) plates containing kanamycin. Although several selections were performed, no kanamycin resistant conjugant was isolated (Fig. S3), indicating that *Xcc fabH* is essential for *Xcc* growth.

### *Xcc fabH* can be replaced with *E. coli fabH*

Bioinformatics analyses showed that *Xcc* FabH is highly identical to *E. coli* FabH. Thus, to test whether a straight chain-specific FabH could provide *Xcc* FabH function, we replaced the *Xcc fabH* with the in-frame *E. coli fabH*. First, we constructed a replacement mutant plasmid, pZTT-3, a pK18mobsacB-borne plasmid that carries a long DNA fragment that includes a 500-bp upstream flanking sequence of *Xcc fabH*, the complete *E. coli fabH* gene, and a 500-bp downstream flanking sequence of *Xcc fabH* (Fig. S4A). After the introduction of pZTT-3 into the wild-type *Xcc* strain 8004 by conjugation, cultures were plated on NYG containing sucrose. Colony PCR assays using the primer pair EcfabH NdeI and EcfabH XbaI (Table S2) showed that one of the colonies, named strain *Xcc* T-3 (*Xcc fabH*::*EcfabH*), contained the 1.3 kb *E. coli fabH*-containing fragment (Fig. S4B,C). As expected no fragments were amplified from the wild type strain using the same primer pair (Fig. S4B,C). The replacement of *Xcc fabH* with *E. coli fabH* in strain *Xcc* T-3 was also confirmed by sequencing of the PCR fragment of the insertion allele.

**Figure 4:**
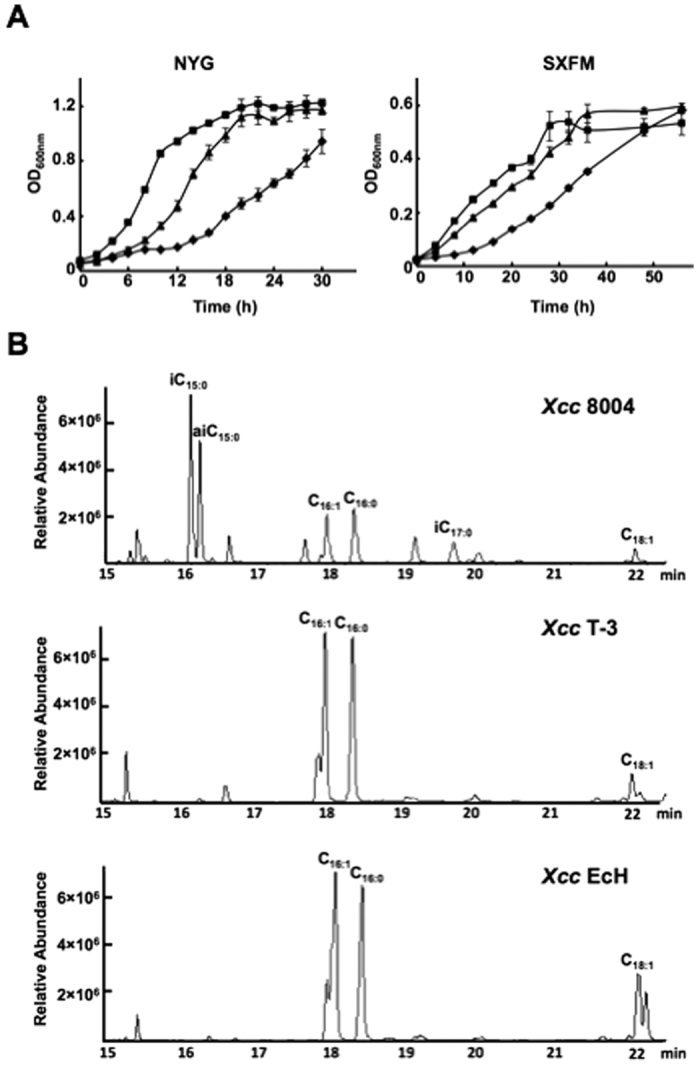
Growth of the *Escherichia coli fabH* replacement mutants in NYG or SXFM medium and fatty acid profiles of the *E. coli fabH* replacement mutants. (**A**) Growth of the *E. coli fabH* replacement mutants in NYG or SXFM medium. Each growth assay was carried out in triplicate in NYG or SXFM at 30 °C and the averages are given. Square indicates *Xcc* wild type strain 8004. The triangle indicates mutant strain *Xcc* EcH. Diamond indicates mutant strain *Xcc* T-3. (**B**) Fatty acid profiles of *Xcc* wild type strain 8004, mutant strain *Xcc* T-3 and *Xcc* EcH. iC_15:0_, 13-methyl-tetradecanoic acid; aiC_15:0_, 12-methyl-tetradecanoic acid; C_16:1_, palmitoleic acid; C_16:0_, palmitic acid; iC_17:0_, 15-methyl-palmitic acid; C_18:1_, *cis*-11-octadecenoic acid.

We then examined the growth of *Xcc* T-3 in NYG or minimal medium (SXFM). The growth of *Xcc* T-3 was substantially slower than that of wild type strain *Xcc* 8004 in either medium (Fig. 4A). To investigate possible reasons for this observation, two possibilities were tested. The first possibility was that the difference of *E. coli* FabH substrate specificities from *Xcc* FabH caused *Xcc* T-3 strain not to produce enough BCFAs to maintain its growth. In fact, *Xcc* T-3 lost the ability to produce BCFAs (Please see next section). Therefore, we supplemented *iso*-C_14:0_, *iso*-C_15:0_, or *anteiso*-C_15:0_ fatty acids into the NYG medium and tested if these fatty acids restored *Xcc* T-3 growth. However, all these long-chain BCFAs failed to restore the growth of strain *Xcc* T-3 in NYG medium (data not shown). This indicated that less production of BCFAs is not the main reason for the weak growth of *Xcc* T-3. The second possibility was that expression of *E.coli* FabH causes the low rate of fatty acid biosynthesis in *Xcc* T-3, which leads to its growth weak. We determined the rate of fatty acid biosynthesis in cell free extracts of *Xcc* T-3 using acetyl-CoA as a substrate and monitored the decrease in NADPH absorbance at 340 nm. The results showed that the rate of fatty acid biosynthesis in cell free extracts of *Xcc* T-3 (47.7 ± 0.37 μmol/mg/min) was much lower than that in cell free extracts of the wild type strain 8004 (60.0 ± 7.39 μmol/mg/min). To further test this possibility, we tried to increase the rate of fatty acid biosynthesis in the *Xcc* strain by overexpression of *E. coli* FabH. We constructed a new replacement mutant by introducing an *E. coli fabH*-encoded plasmid pSRK-EcfabH into the *Xcc fabH* single crossover integrant *Xcc* YH1 (Fig. S5A). After selection, we successfully obtained the *Xcc fabH* deletion strain *Xcc* EcH, in which the *E. coli fabH* gene was expressed from plasmid pSRK-Gm, whereas the *Xcc fabH* gene had been deleted from the genome (Fig. S5B,C). The data showed that the rate of fatty acid biosynthesis in cell free extracts of *Xcc* EcH (67.7 ± 11.36 μmol/mg/minute) was slightly higher than that of the wild type strain. We subsequently tested the growth of strain *Xcc* EcH in both NYG and SXFM media (Fig. 4A). The results showed that strain *Xcc* EcH grew faster than strain *Xcc* T-3 in both media, while the growth of strain *Xcc* EcH was still weaker than that of wild type strain 8004. The expression level of *fabH* in strain *Xcc* 8004, *Xcc* T-3, and *Xcc* EcH was determined by qRT-PCR and data showed that the expression level of *Xcc fabH* in the wild type strain was almost the same as that of *E. coli fabH* in *Xcc* T-3, while the expression level of *E. coli fabH* in *Xcc* EcH was 37-fold higher than that of *E. coli fabH* in *Xcc* T-3 (data not shown). These data suggested that overexpression of *E. coli fabH* in *Xcc* EcH could increase the rate of fatty acid biosynthesis to support growth of the mutant. However, there are other possible causes for the weak growth of the *fabH* mutant, and these possibilities are being further investigated.

### Replacement of *Xcc fabH* with *E. coli fabH* causes loss of *Xcc* ability to synthesize BCFAs

It has been reported that replacement of *S. coelicolor fabH* with *E. coli fabH* leads to a dramatic change in the fatty acid profile of *S. coelicolor*^18^. Although *E. coli fabH* could replace *Xcc fabH*, it was unknown if replacement of *Xcc fabH* with *E. coli fabH* would lead *Xcc* to changes in the fatty acid profile. Thus, the fatty acid composition of strain *Xcc* T-3 grown in NYG medium was determined by gas chromatography-mass spectrometry (GC-MS) (Fig. 4B and Table 2). The fatty acid profiles for wild type strain *Xcc* 8004 consisted of *iso*-BCFAs, *anteiso*-BCFAs, straight-chain saturated fatty acids, and straight-chain unsaturated fatty acids (Fig. 4B and Table 2). The *iso*-BCFAs (46.66 ± 4%) were the predominant fatty acids, including *iso*-C_15:0_ (24.2 ± 0.74%) and *iso*-C_17:1_ (10.68 ± 2.85%). The *anteiso*-BCFAs comprised 17.6 ± 0.48%, mainly of *anteiso*-C_15:0_ (16.77 ± 0.74%). The predominant straight-chain fatty acid was n-C_16:1_ (15.8 ± 0.82%). These results were consistent with a previous report^24^. However, the fatty acid composition of strain *Xcc* T-3 was very different from *Xcc* 8004, being comprised of more than 99% straight-chain fatty acids with only trace amounts of BCFAs (<1%). The predominant fatty acids were n-C_16:1_ (44.44 ± 2.61%), n-C_16:0_ (29.44 ± 1.27%), and n-C_18:1_ (20.11 ± 1.72%) (Fig. 4B and Table 2). We also determined the fatty acid profiles of strain *Xcc* EcH grown in the same medium (Fig. 4B and Table 2). The species and amounts of fatty acids of *Xcc* EcH were almost the same as those of *Xcc* T-3, in which the predominant fatty acids (>99%) were also straight-chain fatty acids, with only trace amounts (<1%) of BCFAs. These data indicated that *Xcc* FabH was a key enzyme in the biosynthesis of BCFAs by *Xcc*.

To confirm that the BCFA synthesis defect in strain *Xcc* T-3 was due to the absence of the *Xcc fabH* gene, complementation analyses were performed. A plasmid, pYYH-1, in which *Xcc fabH* was placed under the control of the isopropyl β-D-1-thiogalactopyranoside-inducible *P*_*lac*_ promoter was introduced into strain *Xcc* T-3. Expression of *Xcc fabH* in strain *Xcc* T-3 (strain *Xcc* YH4) reestablished the synthesis of BCFAs. However, as shown in Table 2, strain *Xcc* YH4 synthesized a smaller amount of BCFAs than the wild-type strain 8004, likely due to the activity of *E. coli* FabH for acetyl-CoA, which was higher than that of *Xcc* FabH.

To further investigate the function of *Xcc* FabH in BCFA synthesis, the fatty acid profiles of strain *Xcc* T-3 grown in SXFM medium were determined by GC-MS (Table S4). As seen for cultures grown in NYG medium, strain *Xcc* T-3 grown in SXFM medium did not produce any detectable BCFAs and contained almost the same species of fatty acids as those grown in NYG medium. Moreover, the fatty acid composition of strain *Xcc* EcH grown in SXFM medium was not significantly different from that grown in NYG medium (Table S4). However, although wild type strain *Xcc* 8004 also had a fatty acid profile similar to that grown in NYG medium, the total amount of BCFAs decreased, and there was a switch between the two species of BCFAs, with *anteiso*-BCFA becoming the major BCFA species (Table S4). These results indicated that the medium composition influences the fatty acid profiles of *Xcc*.

We also tested the effect of temperature on the fatty acid composition of *Xcc* strains grown in SXFM (Table S4). As the temperature dropped from 30 to 15 °C, the wild-type strain 8004 significantly increased the production of n-C_16:1_ fatty acids from 25.54 to 40.44% of the total fatty acids, which resulted in an increase in the total unsaturated fatty acid content from 36.25 to 49.75%. At the same time, the total BCFA content of strain 8004 decreased from 38.34 to 26.37%, especially the *anteiso*-BCFAs, which decreased from 23.34 to 14.83%. These data suggested that the wild-type *Xcc* strain increases the unsaturated fatty acid content, not the *anteiso*-BCFA content, in response to low temperatures. Low temperatures also affected the fatty acid composition of strains *Xcc* EcH and *Xcc* T-3 (Table S4). At low temperatures, the *Xcc* EcH strain also increased its total unsaturated fatty acid content. Although strain *Xcc* T-3 did not change its total unsaturated fatty acid content significantly, its n-C_16:1_ fatty acid content increased.

### Replacement of *Xcc fabH* with *E. coli fabH* abrogates the ability of *Xcc* to produce the 11-Me-C_12_:Δ^2^ signal

**Figure 5:**
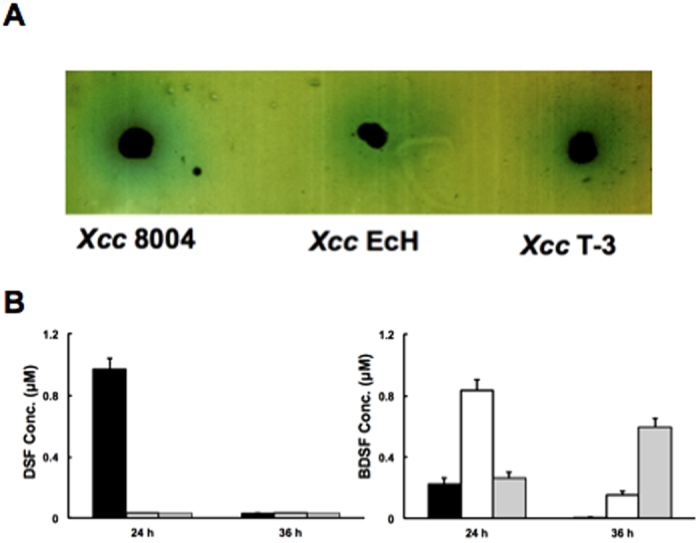
The diffusible signal factor (DSF) family signal produced by *E. coli fabH* replacement mutants. (**A**) DSF-family signal bioassay of *Xcc* mutant strains. The formation of a blue halo due to hydrolysis of 5-bromo-4-chloro-3-indolyl-b-D-glucuronic acid around the site of inoculation indicates the presence of DSF-like activity. (**B**) The amount of DSF-family signal produced by *Xcc* wild type strain 8004, mutant strain *Xcc* EcH and T-3. The black column indicates wild type strain 8004. The white column indicates strain *Xcc* EcH. The gray column indicates strain *Xcc* T-3. Data are means ± the standard deviations of three independent assays.

Previous *in vitro* studies showed that the precursor of C_12_:Δ^2^ was 3-hydroxydodecanoyl-acyl-ACP, and that inhibition of fatty acid biosynthesis using cerulenin, an antibiotic that specifically binds to long-chain 3-keto-acyl-ACP synthases (FabF and FabB), caused *Xcc* to drastically reduce the production of the DSF family signals^8^. These results suggested that the precursors of DSF family signal synthesis come from the fatty acid synthetic pathway. If our hypothesis is correct, the mutant strains, *Xcc* T-3 and *Xcc* EcH, should not produce the 11-Me-C_12_:Δ^2^ signal, because both strains have lost the ability to synthesize BCFAs. To test this hypothesis, we first examined the DSF family signals produced in strains *Xcc* T-3 and *Xcc* EcH by a bioassay using *Xcc* 8523^39^ as a reporter strain. Both *Xcc* T-3 and *Xcc* EcH produced DSF signals (Fig. 5A), indicating that both *Xcc* T-3 and *Xcc* EcH possessed the ability to produce DSF signals. The active fractions of DSF signals produced by mutant strains were separated by high performance liquid chromatography (HPLC). After incubation in NYG medium for 24 hours, the wild type strain 8004 produced mainly 11-Me-C_12_:Δ^2^ (0.97 μM) and C_12_:Δ^2^ (0.23 μM), two DSF signals, while both *Xcc* EcH and *Xcc* T-3 strains only produced the C_12_:Δ^2^ signal (0.83 μM and 0.26 μM, respectively) (Fig. 5B). When the incubation time was increased to 36 hours, both 11-Me-C_12_:Δ^2^ and C_12_:Δ^2^, DSF signals, produced by the wild type strain 8004, declined to trace amounts, and the concentration of C_12_:Δ^2^ signal in the culture of strain *Xcc* EcH decreased to 0.15 μM (Fig. 5B). These results were consistent with a previous report that DSF signals reached a maximum at stationary phase and decreased drastically when bacterial numbers declined^32^. However, at the same time, the amount of C_12_:Δ^2^ in the culture of strain *Xcc* T-3 was increased to 0.6 μM (Fig. 5B), and this was probably due to the low growth rate of *Xcc* T-3, which caused the strain *Xcc* T-3 to enter the stationary phase later than did the wild type strain or *Xcc* EcH strain. Moreover, the data also showed that during these conditions, neither mutant strain produced the 11-Me-C_12_:Δ^2^ signal. These observations confirmed that the precursors of DSF signals come from the intermediates of the fatty acid synthesis pathway.

### Replacement of *Xcc fabH* with *E. coli fabH* leads to reduced *Xcc* virulence in plants

**Figure 6:**
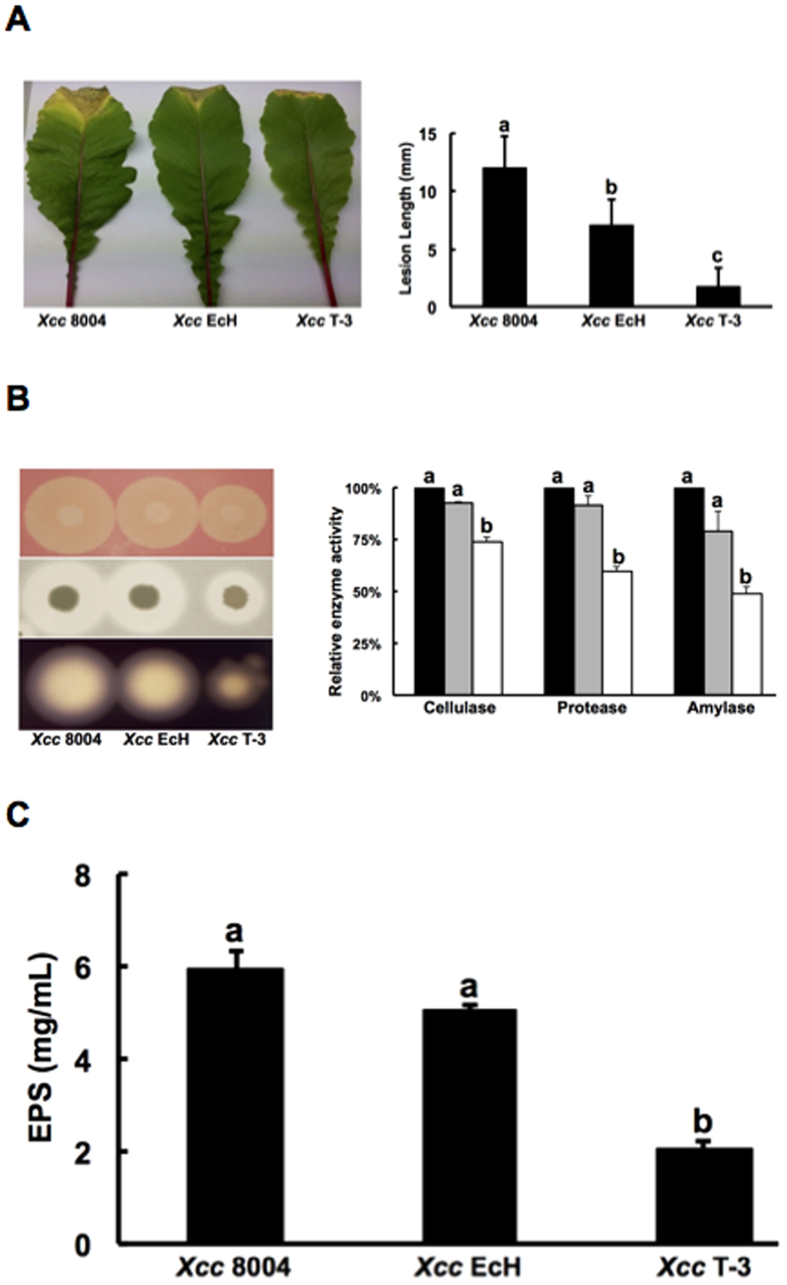
Effects of *Escherichia coli fabH* on the virulence of *Xcc*. (**A**) Pathogenicity test on Chinese radish with the *Xcc* wild type strain 8004, mutant strain *Xcc* EcH and T-3. Virulence of the *Xcc* strains was tested by measuring lesion length after introducing bacteria into the vascular system of Chinese radish by leaf clipping according to the previous report^49^. Values are expressed as the mean and standard deviation of triplicate measurements, each comprised of 10 leaves. Different letters indicate significant differences between treatments (P = 0.05). (**B**) The relative activity of extracellular enzymes produced by *Xcc* strains in NYG. The black columns indicate the *Xcc* wild type strain 8004, the gray columns indicate the mutant strain *Xcc* EcH, and the white columns indicate mutant strain *Xcc* T-3. (**C**) The amount of extracellular polysaccharide (EPS) produced by the *Xcc* strains. Data are the mean ± standard deviation of triplicate measurements. The different letters in each data column indicate significant differences at P = 0.01.

Previous studies confirmed that membrane BCFAs play a key role in virulence regulation of *L. monocytogenes*, which included *anteiso*-BCFA-enhanced bacterial resistance against phagosomal killing by macrophages and modulation of the production of the critical virulence factor, and listeriolysin O^40^,^41^. *Xcc* wild type strain 8004 has the ability to synthesize BCFAs, whereas replacement of *Xcc fabH* with *E. coli fabH* in the mutant strains, *Xcc* EcH or *Xcc* T-3, results in the inability to synthesize BCFAs. To investigate the role of BCFAs in virulence regulation of *Xcc*, the pathogenesis of these mutant strains on plants was tested. A leaf clipping virulence assay using Chinese radish was conducted. The average lesion length caused by the wild type strain 8004 on a leaf of Chinese radish was 12.4 mm after 2 weeks inoculation (Fig. 6A). Replacement of *Xcc fabH* with a single copy of *E. coli fabH* in strain *Xcc* T-3 resulted in a significantly reduced average lesion length (1.7 mm) (Fig. 6A). This suggested that a lack of BCFAs seems to cause a reduction of *Xcc* plant pathogenesis. However, the average lesion length on Chinese radish leaves caused by strain *Xcc* EcH, a strain that carried an *E. coli fabH* encoding plasmid with a chromosomal *fabH* in-frame deletion, was 7.1 mm (Fig. 6A), which was shorter than that caused by the wild type strain 8004, but longer than that caused by strain *Xcc* T-3. Although both mutant strains lost the ability to produce BCFA and had a similar fatty acid profile, the growth rate of *Xcc* T-3 was lower than that of strain *Xcc* EcH. Thus, we speculate that reduction of pathogenesis to Chinese radish leaves caused by *Xcc* T-3 was mainly due to the lower growth rate of *Xcc* T-3, and not due to the lack of BCFAs. To confirm this hypothesis, we determined the growth of these mutant strains in fully mature Chinese cabbage (Wongbok) extracts. The data showed that the growth of strain *Xcc* T-3 was significantly weaker than that of the wild type strain, and the growth of *Xcc* EcH was faster than that of *Xcc* T-3, but was still slower than that of the wild type strain (Fig. S6A). We also determined the CFU in Chinese cabbage after inoculation with the mutant strains. The CFU in Chinese cabbage infected by *Xcc* T-3 was lower than that of those infected by the wild type strain or *Xcc* EcH during 6 days after inoculation (Fig. S6B). Another possible cause could be the difference of the total amount of DSF signals produced among the *Xcc* strains. Although it has been reported that 11-Me-C_12_:Δ^2^ and C_12_:Δ^2^ have almost the same effects on the induction of pathogenesis toward plants^8^, the total amount of DSF signals produced by the *Xcc* T-3 and *Xcc* EcH strains was lower than that produced by the wild-type *Xcc* strain 8004 (Fig. 5B).

We also evaluated several pathogenicity-related virulence factors produced by mutant strains. The activity of extracellular enzymes (including cellulase, amylase, and protease) was first tested. Substitution of *Xcc fabH* with a single copy of *E. coli fabH* in strain *Xcc* T-3 resulted in a significant decrease in the production of extracellular cellulase, amylase, and protease (Fig. 6B). The levels of cellulase, protease, and amylase produced by *Xcc* T-3 were only 74%, 60%, and 49%, respectively, of that produced by the wild-type strain. Although *Xcc* EcH slightly reduced the production of extracellular cellulase, amylase, and protease, the observed decreases were not statistically significant (Fig. 6B). Next, extracellular polysaccharide (EPS) production by mutant strains was tested. The amounts of EPS produced by wild type 8004, mutant strain, *Xcc* EcH or *Xcc* T-3 were 7.1 mg/mL, 5.9 mg/mL, and 2.0 mg/mL, respectively. The EPS produced by *Xcc* T-3 was significantly lower than that produced by wild-type strain 8004, but the amount of EPS produced by *Xcc* EcH was not statistically different from that produced by wild-type strain 8004 (Fig. 6C). These data also support our hypothesis that although replacement of *Xcc fabH* with *E. coli fabH* affects the pathogenesis in host plants, BCFAs do not play a key role in the regulation of *Xcc* virulence.

## Discussion

**Figure 7:**
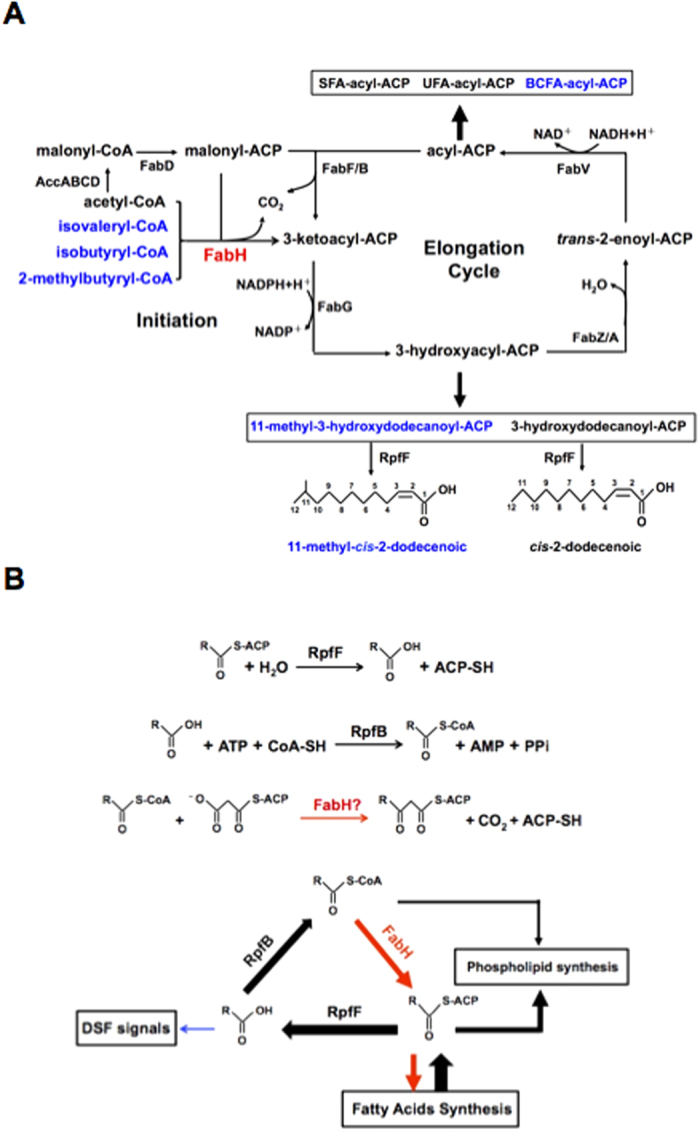
Schematic biosynthetic pathway of fatty acid and DSF family signal in *Xcc* and proposed model for the interplay of RpfF, RpfB, and FabH in *Xcc*. (**A**) The fatty acid and DSF family signal biosynthetic pathway in the *Xcc* strain. Abbreviations: Acc, acetyl-CoA carboxylase; FabD, malonyl-CoA ACP transacylase; FabH, 3-ketoacyl-ACP synthase III; FabG, 3-ketoacyl-ACP reductase; FabZ/A, 3-hydroxyacyl-ACP dehydratase; FabB/F, 3-ketoacyl-ACP synthase I/II; FabV, enoyl-ACP reductase; and, RpfF, acyl-ACP thioesterase/dehydratase. (**B**) Proposed model for interplay of RpfF, RpfB, and FabH in *Xcc*. The reactions catalyzed by RpfF, RpfB, and FabH are shown on the top of the figure. The interplay of RpfF, RpfB, and FabH are shown on the bottom of the figure.

The 3-ketoacyl-ACP synthase III, FabH, condenses acyl-CoAs with malonyl-ACP to initiate fatty acid biosynthesis in the type II fatty acid synthase systems of bacteria^1^,^2^,^4^. *Xcc* produces BCFAs, which account for approximately 50% of the total fatty acids of the *Xcc* cell. Bioinformatics analyses showed that *Xcc* FabH is highly identical to the FabH proteins from straight-chain fatty acid-producing Gram-negative bacteria, such as *E. coli* or *R. solanacearum*, but has a low amino acid sequence homology with the FabH proteins from BCFA synthesizing Gram-positive bacteria, such as *B. subtilis* or *L. monocytogenes*. This suggests that *Xcc* FabH prefers to select acetyl-CoA as a primer to initiate straight-chain fatty acid synthesis rather than to produce BCFAs. However, in the present study, we found that the *Xcc fabH* gene was able to restore the growth of the *R. solanacearum fabH* deletion mutant strain, and it caused this strain to produce certain BCFAs. *In vitro* assays demonstrated that *Xcc* FabH condensed branched-chain acyl-CoAs with malonyl-ACP to initiate fatty acid biosynthesis. Moreover, substitution of *Xcc fabH* with *E. coli fabH* resulted in the loss of *Xcc* ability to synthesize BCFAs. These results demonstrated that *Xcc* FabH is a key enzyme required for *Xcc* to synthesize BCFAs and provided the first evidence that in BCFA-producing Gram-negative bacteria, FabH proteins also prefer to use branched-chain acyl-CoAs as substrates to initiate fatty acid biosynthesis (Fig. 7A).

The *fabH* is an essential gene for growth of *Xcc* and cannot be disrupted from its genome, suggesting that fatty acid biosynthesis is a key metabolic pathway for *Xcc*. However, *Xcc* does not have an obligatory growth requirement for BCFAs. First, the mutant strain *Xcc* T-3, in which *Xcc fabH* was replaced with the *E. coli fabH* gene, lost the ability to synthesize BCFAs, but could grow in either enriched or minimal media, even though it grew slower than the wild-type strain 8004. Second, exogenous *iso*-C_14:0_, *iso*-C_15:0_, or *anteiso*-C_15:0_ did not restore the growth rate of this mutant. Third, the *Xcc* T-3 strain was still pathogenic toward Chinese radish leaves, even though the virulence of this strain was lower than that of the wild-type strain 8004. Moreover, we showed that the fatty acid profiles of another *Xcc fabH* mutant, *Xcc* EcH, which carried an *E. coli fabH*-encoding plasmid and a chromosomal *fabH* in-frame deletion, were similar to those of strain *Xcc* T-3. The *Xcc* EcH strain grew faster in either enriched or minimal media, and was more virulent toward a host plant than strain *Xcc* T-3. These data also demonstrate that BCFAs are not required for *Xcc* growth and pathogenesis.

The *Xcc* mutant strains *Xcc* T-3 and *Xcc* EcH produced more unsaturated fatty acids than did the wild-type strain 8004, indicating that, to some extent, unsaturated fatty acids were able to replace the function of BCFAs in maintaining the membrane fluidity of *Xcc*. In addition, we observed that *Xcc* responds to a decrease in the ambient temperature by increasing the proportion of unsaturated fatty acids, especially n-C_16:1_ fatty acids, in the membrane lipids. This adaptive response markedly differs from that in *L. monocytogenes*, which mainly stimulates *anteiso*-BCFA biosynthesis to increase membrane disorder^40^,^41^. Although the mechanism of unsaturated fatty acid biosynthesis in *Xcc* has not been well studied, the *Xcc* genome encodes two key anaerobic, unsaturated fatty acid biosynthesis genes, *fabA* (XC_3651) and *fabB* (XC_3652), which could be co-transcribed in a *fabAB* operon, as well as an aerobic, unsaturated fatty acid biosynthesis gene, *desA* (XC_0171). These data suggest that *Xcc* has the ability to produce unsaturated fatty acids, and that unsaturated fatty acids can play a key role in the regulation of membrane fluidity.

Although it has been reported that the inhibition of fatty acid biosynthesis causes *Xcc* to drastically reduce the production of 11-Me-C_12_:Δ^2^ and C_12_:Δ^2^ signals^8^, there is still a lack of *in vivo* evidence to support the view that *Xcc* shunts intermediates from the fatty acid synthesis pathways to produce DSF signals. Our report provides direct *in vivo* evidence that the precursors of DSF signals come from the fatty acid synthesis pathway of *Xcc* (Fig. 7A). Substitution of *Xcc fabH* with *E. coli fabH* not only caused *Xcc* to fail to produce BCFAs, but it also abrogated the synthesis of the 11-Me-C_12_:Δ^2^ signal.

*Xcc* FabH was found to utilize a wide range of substrates as the acyl-CoA primer *in vitro*, but it remains unclear whether *Xcc* FabH exhibits a wide range of substrate specificities *in vivo*. However, we speculate that *Xcc* FabH should possess this feature. In *Xcc*, RpfF has been reported to have a wide range of acyl-ACP thioesterase activities, with the ability to hydrolyze acyl-ACP intermediates of fatty acid synthesis pathways to produce free fatty acids^8^,^9^. RpfB is a fatty acyl-CoA ligase, which converts free fatty acids produced by RpfF to fatty acyl-CoAs. It has been reported that long-chain fatty acyl-CoAs produced by RpfB are converted to phospholipids^42^. But it is unknown how *Xcc* utilizes the rest of the short- or medium-chain fatty acyl-CoAs. One possibility is that *Xcc* FabH condenses the short- or medium-chain fatty acyl-CoAs produced by RpfB with malonyl-ACP to produce new acyl-ACP intermediates, which re-enter the fatty acid synthetic cycle to synthesize long acyl-ACPs (Fig. 7B). This possibility is currently being studied in our laboratory.

## Materials and Methods

### Materials

Malonyl-CoA, acetyl-CoA, acyl-CoAs, fatty acids, NADH, NADPH, and antibiotics were purchased from Sigma-Aldrich. Takara Biotechnology Co. provided molecular biology reagents. Novagen provided the pET vectors. Ni-agarose columns were obtained from Invitrogen. GE healthcare provided the HiTrap Q strong anion-exchange column and Bio-Rad provided the Quick Start Bradford dye reagent. All other reagents were of the highest available quality. Takara Biotechnology Co. synthesized the oligonucleotide primers.

### Bacterial strains, plasmids, and growth conditions

Strains and plasmids used in this study are listed in Table S1. *E. coli* strains were grown in Luria–Bertani medium at 37 °C. *Xcc* strains were grown at 30 °C in NYG medium (5 g/L tryptone, 3 g/L yeast extract, and 20 g/L glycerol)^8^ or SXFM (0.7 g/L K_2_HPO_4_; 0.2 g/L KH_2_PO_4_; 1 g/L (NH_4_)_2_SO_4_; 0.1 g/L MgCl_2_; 0.01 g/L FeSO_4_; 0.001 g/L MnCl_2_; 0.625 g/L yeast extract; and 10 g/L sucrose), and 40 μg/mL histidine, 40 μg/mL aspartic acid, and 40 μg/mL glutamic acid were added to the SXFM. When required, antibiotics were added at the following concentrations: 100 μg/mL sodium ampicillin; 30 μg/mL kanamycin sulfate; 30 μg/mL gentamycin for *E. coli* or 10 μg/mL for *Xcc*; and 50 μg/mL rifampicin. Bacterial growth in liquid medium was determined by measuring optical density at 600 nm (OD_600_).

We also tested the growth of *Xcc* strains in cabbage extracts. The cabbage juice was prepared as follows. Mature Chinese cabbage (Wongbok) (0.5 kg) was minced using an electric juicer and the supernatant was collected by centrifugation at room temperature. Then, the supernatant was filtered using a membrane filtration system (0.22 μm) to obtain the cabbage extracts subsequently used for growth analyses.

### Complementation of the *R. solanacearum fabH* deletion strain

The *Xcc fabH* gene was amplified from genomic DNA of *Xcc* wild type strain 8004 using primers listed in Table S2. Then the amplified fragment was purified, digested with NdeI and HindIII, and cloned into the same sites of pSRK-Km^38^ to get plasmid pYYH-1. Following the mating of derivatives of *E. coli* strain S17-1 carrying the pYYH-1 or empty vector, with *R. solanacearum fabH* deletion strain RsmH^35^ on BG plates with octanoic acid (0.1%) for 48 hours at 30 °C, the cells were suspended in BG medium, and appropriate dilutions were inoculated onto BG plates (with octanoic acid) containing chloramphenicol (to select against the donor strain) plus kanamycin. The transformed strains were inoculated onto the BG plates with or without octanoic acid, and growth was determined after 2 days incubation at 30 °C.

### Protein expression and purification

The *Xcc fabH* gene was cloned into pET-28b to yield plasmid pYYH-2. *Xcc* FabH with a vector-encoded His_6_-tagged N-terminus was expressed in *E. coli* BL21 (DE3), and purified with Ni-NTA agarose (Qiagen) using a nickel-ion affinity column (Qiagen). The purities of proteins were monitored by SDS-PAGE. The *E. coli* FabD, FabH, FabG, FabZ, and FabI, and *E. coli* holo-ACP proteins were purified as described previously^43^.

### Assay of 3-ketoacyl-ACP synthase activities *in vitro*

The assay mixture contained 0.1 M sodium phosphate (pH 7.0); 0.1 μg each of EcFabD, FabH (XccFabH or EcFabH), EcFabG, and EcFabZ; 50 μM NADH; 50 μM NADPH; 1 mM β-mercaptoethanol; 100 μM malonyl-CoA; 50 μM holo-ACP; and 100 μM of substrate (acetyl-CoA, butyryl-CoA, isobutyryl-CoA, isovaleryl-CoA, hexanoyl-CoA, octanoyl-CoA, capryl-CoA, or lauryl-CoA) in a final volume of 40 μL. The reactions were initiated by addition of FabH and followed by incubation for 1 hour. The reaction products were resolved by conformationally sensitive gel electrophoresis on 20% polyacrylamide gels containing a concentration of urea optimized for the separation^44^. The gels were stained with Coomassie Brilliant Blue R-250.

### Spectrophotometric assay of 3-ketoacyl-ACP synthase activity

A continuous assay format was used to monitor *Xcc* FabH activity with straight- and branched-chain acyl-CoA by coupling the condensing activity of *Xcc* FabH to 3-ketoacyl-ACP reductase (FabG) of *E. coli* as described previously^45^. The reaction mixture contained 100 μM ACP, 0.5 mM acyl-CoA, 0.5 mM malonyl-CoA, 0.2 mM NADPH, 2 μg of FabG, 2 μg of purified *E. coli* FabD, and 0.1 M sodium phosphate buffer, pH 7.4, in a final volume of 100 μL. The reaction was initiated by adding 1 μg *Xcc* FabH to the mixture, and the 3-ketoacyl-ACP synthase III activities of *Xcc* FabH were determined by monitoring the rate of oxidation of NADPH at 340 nm using an extinction coefficient of 6,220 M^−1^.

### Disruption of the *Xcc fabH* gene

To disrupt the *Xcc fabH* gene, a pK18mobsacB-borne in-frame deletion suicide plasmid, pZTT-1, was constructed according to Fig. S2A. The 500 bp DNA fragments flanking up or down the *Xcc fabH* gene were amplified with *Pfu* DNA polymerase using *Xcc* genomic DNA as the template, and either XcfabH1 EcoRI and XcfabH2 (for up fabH), or XcfabH3 and XcfabH4 HindIII (for down fabH), were used as primers (Table S2). The two fragments were purified and joined by overlapping PCR. The fused fragment was then digested with EcoRI and HindIII, and inserted between the same sites of pK18mobscaB^46^ to give the plasmid pZTT-1. We also constructed a suicide plasmid, pYYH-3, in which a 550 bp inner DNA fragment of *Xcc fabH* was inserted into plasmid pK18mobsacB (Fig. S3). Following the mating of derivatives of *E. coli* strain S17-1 carrying suicide plasmid (pZTT-1 or pYYH-3) with *Xcc* 8004 on NYG plates for 36 hours at 30 °C, the cells were suspended in NYG medium, and appropriate dilutions were inoculated onto NYG plates containing rifampicin (to select against the donor strain) plus kanamycin to select for the integration of the non-replicating plasmid into the chromosome of the recipient. Single crossover integrants of pZTT-1 (i.e., *Xcc* YH1) (Fig. S2) were selected for further study, while no integrant of pYYH-3 was obtained (Fig. S3). Then the culture of *Xcc* YH1 was incubated at 30 °C for 36 hours, and after appropriate dilutions, the culture was inoculated onto NYG plates containing 10% sucrose. Colonies sensitive to kanamycin were screened by colony PCR with primers listed in Table S2. NYG medium with fatty acid supplementation^47^ was also used to select *Xcc fabH* deletion mutants.

### Replacement of *Xcc fabH* with *E. coli fabH*

To replace *Xcc fabH* in the chromosome, the *E. coli fabH* gene (*EcfabH*) was amplified from *E. coli* MG1655 genomic DNA with *Pfu* DNA polymerase and the primers listed in Table 2S. The *EcfabH* gene was ligated into the T-vector plasmid pMD19 to yield pZTT-2. The NdeI-BamHI fragment of *EcfabH* was cloned into the same sites of pZTT-1 to yield suicide plasmid pZTT-3. *E. coli* strain S17-1 carrying plasmid pZTT-3 was conjugated with *Xcc* 8004 on NYG plates for 24 hours at 30 °C (Fig. S4A). After appropriate dilutions, the colonies, which were resistant to rifampicin and kanamycin, were selected, and one of these colonies was named *Xcc* YH2. *Xcc* YH2 was inoculated into NYG medium at 30 °C for 48 hours, and the cultures were spread onto NYG plates containing 10% sucrose. Colony PCR utilizing the primers listed in Table S2 was performed to screen colonies sensitive to kanamycin. We successfully obtained strain *Xcc* T-3 (Fig. S4B,C), in which *Xcc fabH* was replaced with *E. coli fabH* in the chromosome.

Another replacement of *Xcc fabH* with *E. coli fabH* strain, *Xcc* EcH, was also constructed as follows (Fig. S5A). First, *EcfabH* encoding plasmid pSRH-*EcfabH* was introduced into the *Xcc fabH* single crossover integrant *Xcc* YH1 to obtain *Xcc* YH5 by conjugation with *E. coli* S17-1 (Fig. S5A). The culture of *Xcc* YH5 was inoculated onto NYG plates containing 10% sucrose. Colonies sensitive to kanamycin were screened by colony PCR using the primers listed in Table S2. One colony, in which plasmid pSRK-*EcfabH* expressed *E. coli fabH* and *Xcc fabH* had been deleted from the chromosome, was obtained, and it was named *Xcc* EcH (Fig. S5B,C).

### Analysis of fatty acid composition

The cultures of bacteria were grown aerobically in different media for 2–4 days. Cells were harvested and washed three times with water. Fatty acid methyl esters were synthesized and extracted as described previously^24^. Briefly, cellular lipids were saponified by addition of 1 mL of sodium hydroxide/methanol solution at 100 °C for 40 minutes with shaking (800 rpm). The fatty acids were then methylated by addition of 2 mL of hydrochloric acid/methanol solution at 80 °C for 30 minutes, and immediately cooled to below 20 °C. Fatty acid methyl esters were obtained by three extractions each with 1 mL of petroleum ether. The solvent was removed under a stream of nitrogen, and the residue was analyzed by GC-MS.

### Extraction and purification of DSF-family signal components from the *Xcc* culture supernatant

The protocol for extraction and purification of DSF family components was described previously^48^. In brief, *Xcc* strains were cultured in liquid medium for 24–48 hours and 50 mL of bacterial supernatant was collected by centrifugation at 3,800 × g for 30 minutes at 4 °C. The pH of the supernatants was adjusted to 4.0 by adding hydrochloric acid prior to two extractions with an equal volume of ethyl acetate. The ethyl acetate fractions were collected and the solvent was removed by rotary evaporation to dryness at 40 °C. The residue was dissolved in 1 mL of methanol. The crude extract was subjected to a 0.45 μm Minisart filter unit and the collected filtrate was concentrated to 0.5 mL. Three microliters of the extract was injected into a C18 reverse-phase HPLC column (4.6 × 150 mm, Agilent Technologies), eluted with water in methanol (23:77, v/v, respectively; 0.1% formic acid) at a flow rate of 1 mL/minute in an Agilent Technologies 1260 Infinity system with a DAD G1315D VL detector.

### Bioassay and quantitative analyses of DSF family signaling components

The DSF bioassay was performed as described previously^32^. 11-Me-C_12_:Δ^2^ and C_12_:Δ^2^ production were quantified using peak area (A) of the HPLC eluant by the following formula: 11-Me-C_12_:Δ^2^ (μM) = 1.32A–50.24 and C_12_:Δ^2^ (μM) = 0.71A–10.64. The formula was derived from a dose-peak area plot of the HPLC eluant using various dilutions of synthetic 11-Me-C_12_:Δ^2^ and C_12_:Δ^2^ components with a correlation coefficient (R^2^) of 0.999 and 0.998, respectively.

### Pathogenicity tests

Virulence was tested on potted Chinese radish (*Raphanus sativus* L. var. radiculus Pers.) as described previously^49^. Bacterial growth in mature Chinese cabbage (Wongbok) was tested as follows. Bacterial cells were grown in NYG medium at 30 °C for 24 h, then collected by centrifugation and washed twice with sterile saline. The cell pellets were suspended in sterile saline to a final OD_600_ of 0.05. Cabbage stem discs were sampled using a cork borer (diameter, 14.25 mm), and the discs were immersed in bacterial suspension for 2 h. After washing twice with sterile water, the discs were kept in empty plates at 25 °C, and ground with a pestle in 1 ml sterile water at day 0, 3, 6, and 9 after inoculation. The homogenates were serially diluted, and dilutions were plated on NYGA supplemented with rifampicin. Bacterial colony-forming units were counted after incubation at 30 °C for 3 days.

### Measurement of extracellular enzymatic activity and EPS production

Relative activities of extracellular enzymes were assayed as described previously^50^. Two μL of each *Xcc* strain culture (OD_600_ ≈ 1.0) was spotted onto NYG agar plates containing 1% (w/v) skim milk (for protease), 0.5% (w/v) carboxymethylcellulose (for cellulase), or 0.1% (w/v) starch (for amylase) and incubated at 30 °C for 24–48 hours. Plates were stained where necessary as previously described^50^. Zones of clearance around the spot due to the degradation of the substrate were photographed. Three plates were inoculated in each experiment, and each experiment was repeated three times. The relative activity of the enzyme was indicated by the diameter of the clear zone.

The EPS production was measured as described previously^51^. Each *Xcc* strain culture (2 mL, OD_600_ ≈ 1.0) was used to inoculate 100 mL of NYG containing 4% glucose in a 250 mL flask and kept at 30 °C with shaking at 180 rpm for 4 days. The EPS was precipitated from the culture supernatant by addition of 4 volumes of ethanol. The pelleted EPS was washed with 70% ethanol, air dried, and weighed. Three flasks were inoculated in each experiment and each experiment was repeated three times.

### Statistical analyses

Analysis of variance for experimental datasets was performed using JMP software, version 5.0 (SAS Institute Inc., Cary, NC, USA). Significant effects of treatment were determined by the F value (P = 0.05). When a significant F test was obtained, separation of means was accomplished by Fisher’s protected LSD (least significant difference) at P ≤ 0.05.

## Additional Information

**How to cite this article**: Yu, Y.-H. *et al*. *Xanthomonas campestris* FabH is required for branched-chain fatty acid and DSF-family quorum sensing signal biosynthesis. *Sci. Rep.* **6**, 32811; doi: 10.1038/srep32811 (2016).

## Supplementary Material

Supplementary Information

## Figures and Tables

**Table 1:** *Xcc*FabH activity with various acyl-CoAs.

Substrate	*Xcc*FabH activity (μM/μg/min)α
Acetyl-CoA	7.1 ± 1.35e
Isobutyryl-CoA	11.35 ± 0.76d
Isovaleryl-CoA	17.35 ± 1.69c
Butyryl-CoA	46.05 ± 5.62a
Hexanoyl-CoA	33.38 ± 2.56b
Octanoyl-CoA	21.47 ± 3.13c
Decanoyl-CoA	6.72 ± 2.43e
Dodecanoyl-CoA	0.25 ± 0.05f

^α^The values are the means ± standard deviations of three independent experiments. The statistical analyses were performed using Microsoft Excel with *P* values between each pairwise comparison calculated by two-tailed Student *t* tests. Significant differences are indicated by different letters (*P* < 0.05).

**Table 2:** Fatty acid composition of total lipid extracts from *Xcc* 8004 and mutant strains grown on NYG medium^a^.

Fatty acids	Composition (%)			
	*Xcc* 8004	*Xcc* EcH	*Xcc* T-3	*Xcc* YH4
*iso*-C_14:0_^b^	0.92 ± 0.04	0.11 ± 0.09	0.13 ± 0.11	0.60 ± 0.08
n-C_14:0_	1.11 ± 0.10	5.88 ± 1.12	3.05 ± 0.24	3.39 ± 0.89
*iso*-C_15:0_	24.2 ± 0.74	0.12 ± 0.11	0.05 ± 0.08	14.11 ± 1.09
*anteiso*-C_15:0_	16.77 ± 0.42	0.04 ± 0.08	0.03 ± 0.05	13.23 ± 0.96
n-C_15:0_	3.19 ± 0.73	3.82 ± 0.39	0.33 ± 0.03	4.77 ± 0.35
*iso*-C_16:0_	5.32 ± 0.21	0.07 ± 0.06	0	2.18 ± 0.13
n-C_16:1_	15.8 ± 0.82	49.4 ± 3.81	44.44 ± 2.61	26.24 ± 2.10
n-C_16:0_	7.12 ± 0.32	28.9 ± 1.56	29.44 ± 1.27	18.06 ± 1.42
*iso*-C_17:1_	10.68 ± 2.85	0	0	0
*iso*-C_17:0_	5.55 ± 0.16	0	0	2.48 ± 0.32
*anteiso*-C_17:0_	0.82 ± 0.06	0	0.36 ± 0.07	1.23 ± 0.15
n-C_17:0_ cyclo	3.14 ± 0.41	2.81 ± 0.34	0.53 ± 0.09	3.39 ± 0.33
n-C_18:2_	0.47 ± 0.11	0.64 ± 0.23	0.44 ± 0.03	1.02 ± 0.09
n-C_18:1_	2.91 ± 0.44	6.82 ± 0.92	20.11 ± 1.72	7.79 ± 0.74
n-C_18:0_	0.64 ± 0.03	1.03 ± 0.33	1.11 ± 0.13	1.50 ± 0.21
**Total UFAs**	33.01 ± 4.64	59.67 ± 5.31	65.51 ± 4.46	38.45 ± 4.78
**Total BCFAs**	64.26 ± 4.49	0.34 ± 0.34	0.57 ± 0.31	33.82 ± 4.52
**Iso-BCFAs**	46.66 ± 4.00	0.3 ± 0.26	0.18 ± 0.2	19.36 ± 1.79
**Anteiso-BCFAs**	17.6 ± 0.48	0.04 ± 0.08	0.39 ± 0.12	14.46 ± 1.03

^a^Cells were grown in NYG medium for 36 h at 28 °C. The total lipids were extracted and trans-esterified to obtain fatty acid methyl esters, and the products were identified by GC-MS. The values are percentages of total fatty acids and are the means ± the standard deviations of three independent experiments.

^b^n-C_14:0_, 3-tetradecanoic; *iso*-C_15:0_, 13-methyl-tetradecanoic acid; *anteiso*-C_15:0_, 12-methyl-tetradecanoic acid; n-C_15:0_, pentadecanoic acid; 3-OH-C_14:0_, 3-hydroxytetradecanoic; *iso*-C_16:0_, 14-methyl-pentadecanoic acid; n-C_16:1_, *cis*-9-hexadecenoic acid; n-C_16:0_, hexadecanoic acid; *iso*-C_17:0_, 15-methyl-hexadecanoic acid; *anteiso*-C_17:0_, 14-methyl-hexadecanoic acid; n-C_17:0_ cyclo, 9,10-methylene hexadecanoic acid; n-C_18:2_, *cis*-11-*cis*-9-octadecenoic; n-C_18:1_, *cis*-11-octadecenoic acid; n-3-C_18:0_, octadecanoic acid. UFA indicates unsaturated fatty acid; BCFA indicates branch-chain fatty acid.

## References

[b1] Zhang Y. M. & Rock C. O. Membrane lipid homeostasis in bacteria. Nat Rev Microbiol. 6, 222–233 (2008). 18264115 10.1038/nrmicro1839

[b2] White S. W. . The structural biology of type II fatty acid biosynthesis. Annu Rev Biochem. 74, 791–831 (2004). 15952903 10.1146/annurev.biochem.74.082803.133524

[b3] Heath R. J., White S. W. & Rock C. O. Lipid biosynthesis as a target for antibacterial agents. Prog Lipid Res. 40, 467–497 (2001). 11591436 10.1016/s0163-7827(01)00012-1

[b4] Campbell J. W. & Cronan J. E. Bacterial fatty acid biosynthesis: targets for antibacterial drug discovery. Annu Rev Microbiol. 55, 305–332 (2001). 11544358 10.1146/annurev.micro.55.1.305

[b5] Cronan J. E., Zhao X. & Jiang Y. Function, attachment and synthesis of lipoic acid in *Escherichia coli*. Adv Microb Physiol. 50, 103–146 (2005). 16221579 10.1016/S0065-2911(05)50003-1

[b6] Lin S. & Cronan J. E. The BioC O-methyltransferase catalyzes methyl esterification of malonyl-acyl carrier protein, an essential step in biotin synthesis. J Biol Chem. 87, 37010–37020 (2012). 22965231 10.1074/jbc.M112.410290 PMC3481302

[b7] Lin S. & Cronan J. E. Closing in on complete pathways of biotin biosynthesis. Mol Biosyst. 7, 1811–1821 (2011). 21437340 10.1039/c1mb05022b

[b8] Zhou L. . The multiple DSF-family QS signals are synthesized from carbohydrate and branched-chain amino acids via the FAS elongation cycle. Sci Rep. 5, 13294 (2015). 26289160 10.1038/srep13294 PMC4542539

[b9] Bi H. . The *Burkholderia cenocepacia* BDSF quorum sensing fatty acid is synthesized by a bifunctional crotonase homologue having both dehydratase and thioesterase activities. Mol Microbiol. 83, 840–855 (2012). 22221091 10.1111/j.1365-2958.2012.07968.x PMC3276249

[b10] Parsek M. R. . Acyl homoserine-lactone quorum-sensing signal generation. Proc Natl Acad Sci. 96, 4360–4365 (1999). 10200267 10.1073/pnas.96.8.4360 PMC16337

[b11] Val D. L. & Cronan J. E. *In vivo* evidence that S-adenosylmethionine and fatty acid synthesis intermediates are the substrates for the LuxI family of autoinducer synthases. J Bacteriol. 180, 2644–2651 (1998). 9573148 10.1128/jb.180.10.2644-2651.1998 PMC107215

[b12] Lu Y. J., Zhang Y. M. & Rock C. O. Product diversity and regulation of type II fatty acid synthases. Biochem Cell Biol. 82, 145–155 (2004). 15052334 10.1139/o03-076

[b13] Chazarreta Cifre L. . Exploring the biosynthesis of unsaturated fatty acids in *Bacillus cereus* ATCC 14579 and functional characterization of novel acyl-lipid desaturases. Appl Environ Microbiol. 79, 6271–6279 (2013). 23913431 10.1128/AEM.01761-13 PMC3811188

[b14] Singh V. K. . Insertional inactivation of branched-chain α-keto acid dehydrogenase in *Staphylococcus aureus* leads to decreased branched-chain membrane fatty acid content and increased susceptibility to certain stresses. Appl Environ Microbiol. 74, 5882–5890 (2008). 18689519 10.1128/AEM.00882-08 PMC2565972

[b15] Giotis E. S. . Role of branched-chain fatty acids in pH stress tolerance in *Listeria monocytogenes*. Appl Environ Microbiol. 73, 997–1001 (2007). 17114323 10.1128/AEM.00865-06 PMC1800763

[b16] Revill W. P. . β-Ketoacyl acyl carrier protein synthase III (FabH) is essential for fatty acid biosynthesis in *Streptomyces coelicolor* A3 (2). J Bacteriol. 183, 3526–3530 (2001). 11344162 10.1128/JB.183.11.3526-3530.2001 PMC99652

[b17] Singh A. K. . FabH selectivity for anteiso branched-chain fatty acid precursors in low-temperature adaptation in *Listeria monocytogenes*. FEMS Microbiol Lett. 301, 188–192 (2009). 19863661 10.1111/j.1574-6968.2009.01814.x PMC2818224

[b18] Li Y., Florova G. & Reynolds K. A. Alteration of the fatty acid profile of *Streptomyces coelicolor* by replacement of the initiation enzyme 3-ketoacyl acyl carrier protein synthase III (FabH). J Bacteriol. 187, 3795–3799 (2005). 15901703 10.1128/JB.187.11.3795-3799.2005 PMC1112031

[b19] Choi K. H., Heath R. J. & Rock C. O. β -ketoacyl-acyl carrier protein synthase III (FabH) is a determining factor in branched-chain fatty acid biosynthesis. J Bacteriol. 182, 365–370 (2000). 10629181 10.1128/jb.182.2.365-370.2000 PMC94284

[b20] Kaneda T. Iso-and anteiso-fatty acids in bacteria: biosynthesis, function, and taxonomic significance. Microbiol Rev. 55, 288–302 (1991). 1886522 10.1128/mr.55.2.288-302.1991 PMC372815

[b21] He Y. W. & Zhang L. H. Quorum sensing and virulence regulation in *Xanthomonas campestris*. FEMS Microbiol Rev. 32, 842–857 (2008). 18557946 10.1111/j.1574-6976.2008.00120.x

[b22] Vauterin L., Yang P. & Swings J. Utilization of fatty acid methyl esters for the differentiation of new *Xanthomonas species*. Int J Syst Bacteriol. 46, 298–304 (1996).

[b23] Yang P. . Application of fatty acid methyl esters for the taxonomic analysis of the genus *Xanthomonas*. Syst Appl Microbiol. 16, 47–71(1993).

[b24] Stead D. E. Grouping of *Xanthomonas campestris pathovars* of cereals and grasses by fatty acid profiling. EPPO Bull. 19, 57–68 (1989).

[b25] Doan T. T. . Crystallization and preliminary X-ray crystallographic analysis of beta-ketoacyl-ACP synthase I (XoFabB) from *Xanthomonas oryzae* pv. *oryzae*. Acta Crystallogr Sect F Struct Biol Cryst Commun. 67, 1548–1550 (2011). 10.1107/S1744309111040590 PMC3232136 22139163

[b26] Huynh K. H. . Cloning, expression, crystallization and preliminary X-ray crystallographic analysis of beta-ketoacyl-ACP synthase III (FabH) from *Xanthomonas oryzae* pv. *oryzae*. Acta Crystallogr Sect F Struct Biol Cryst Commun. 65: 460–462 (2009). 10.1107/S1744309109009555 PMC2675584 19407376

[b27] Jung J. W. . Cloning, expression, crystallization and preliminary X-ray crystallographic analysis of malonyl-CoA-acyl carrier protein transacylase (FabD) from *Xanthomonas oryzae pv. oryzae*. Acta Crystallogr Sect F Struct Biol Cryst Commun. 64, 1143–1145 (2008). 10.1107/S1744309108035331 PMC2593694 19052370

[b28] Li H. . Determination of the crystal structure and active residues of FabV, the enoyl-ACP reductase from *Xanthomonas oryzae*. Plos One. 6, e26743 (2011). 22039545 10.1371/journal.pone.0026743 PMC3198815

[b29] Ryan R. P. & Dow J. M. Communication with a growing family: diffusible signal factor (DSF) signaling in bacteria. Trends Microbiol. 19, 145–152(2011). 21227698 10.1016/j.tim.2010.12.003

[b30] Deng Y. . Diffusible signal factor family signals provide a fitness advantage to *Xanthomonas campestris* pv. *campestris* in interspecies competition. Environ Microbiol. 18, 1534–1545 (2016). 26913592 10.1111/1462-2920.13244

[b31] Deng Y. . The host plant metabolite glucose is the precursor of diffusible signal factor (DSF) family signals in *Xanthomonas campestris*. Appl Environ Microbiol. 81, 2861–2868 (2015). 25681189 10.1128/AEM.03813-14 PMC4375329

[b32] Wang L. H. . A bacterial cell-cell communication signal with cross-kingdom structural analogues. Mol Microbiol. 51, 903–912 (2004). 14731288 10.1046/j.1365-2958.2003.03883.x

[b33] Qian W. . Comparative and functional genomic analyses of the pathogenicity of phytopathogen *Xanthomonas campestris* pv. *campestris*. Genome Res. 15, 757–767(2005). 15899963 10.1101/gr.3378705 PMC1142466

[b34] Heath R. J. & Rock C. O. Inhibition of β-ketoacyl-acyl carrier protein synthase III (FabH) by acyl-acyl carrier protein in *Escherichia coli*. J Biol Chem. 271, 10996–11000 (1996). 8631920 10.1074/jbc.271.18.10996

[b35] Mao Y. H. . *Ralstonia solanacearum* RSp0194 encodes a novel 3-keto-acyl carrier protein synthase III. Plos One. 10, e0136261 (2015). 26305336 10.1371/journal.pone.0136261 PMC4549310

[b36] Stead D. E. Grouping of plant-pathogenic and some other *Pseudomonas* spp. by using cellular fatty acid profiles. Int J Syst Bacteriol. 42, 281–295 (1992).

[b37] Zhu K. . Precursor and temperature modulation of fatty acid composition and growth of *Listeria monocytogenes* cold-sensitive mutants with transposon-interrupted branched-chain α-keto acid dehydrogenase. Microbiology. 151, 615–623 (2005). 15699210 10.1099/mic.0.27634-0

[b38] Khan S. R. . Broad-host-range expression vectors with tightly regulated promoters and their use to examine the influence of TraR and TraM expression on Ti plasmid quorum sensing. Appl Environ Microbiol. 74, 5053–5062 (2008). 18606801 10.1128/AEM.01098-08 PMC2519271

[b39] Barber C. . A novel regulatory system required for pathogenicity of *Xanthomonas campestris* is mediated by a small diffusible signal molecule. Mol Microbiol. 24, 555–566 (1997). 9179849 10.1046/j.1365-2958.1997.3721736.x

[b40] Sun Y. & O’Riordan M. X. Branched-chain fatty acids promote *Listeria monocytogenes* intracellular infection and virulence. Infect Immun. 78, 4667–4673 (2010). 20823206 10.1128/IAI.00546-10 PMC2976352

[b41] Sun Y. . Fatty acids regulates stress resistance and virulence factor production for *Listeria monocytogenes*. J Bacteriol. 194, 5274–5284 (2012). 22843841 10.1128/JB.00045-12 PMC3457240

[b42] Bi H. . *Xanthomonas campestris* RpfB is a fatty acyl-CoA ligase required to counteract the thioesterase activity of the RpfF diffusible signal factor (DSF) synthase. Mol Microbiol. 93, 262–275 (2014). 24866092 10.1111/mmi.12657 PMC4114240

[b43] Zhu L. . Triclosan resistance of *Pseudomonas aeruginosa* PAO1 is due to FabV, a triclosan-resistant enoyl-acyl carrier protein reductase. Antimicrob Agents Chemother. 54, 689–698 (2010). 19933806 10.1128/AAC.01152-09 PMC2812149

[b44] Zhu L. . The two functional enoyl-acyl carrier protein reductases of *Enterococcus faecalis* do not mediate triclosan resistance. mBio. 4, e00613–e00613 (2013). 24085780 10.1128/mBio.00613-13 PMC3791895

[b45] Qiu X. . Crystal structure and substrate specificity of the beta-ketoacyl-acyl carrier protein synthase III (FabH) from *Staphylococcus aureus*. Protein Sci. 14, 2087–2094 (2005). 15987898 10.1110/ps.051501605 PMC2279320

[b46] Schafer A. . Small mobilizable multi-purpose cloning vectors derived from the *Escherichia coli* plasmids pK18 and pK19: selection of defined deletions in the chromosome of *Corynebacterium glutamicum*. Gene. 145, 69–73 (1994). 8045426 10.1016/0378-1119(94)90324-7

[b47] Lai C. Y. & Cronan J. E. β-ketoacyl-acyl carrier protein synthase III (FabH) is essential for bacterial fatty acid synthesis. J Biol Chem. 278, 51494–51503 (2003). 14523010 10.1074/jbc.M308638200

[b48] He Y. W. . Rice bacterial blight pathogen *Xanthomonas oryzae* pv. *oryzae* produces multiple DSF-family signals in regulation of virulence factor production. BMC Microbiol. 10, 187 (2010). 20615263 10.1186/1471-2180-10-187 PMC2909994

[b49] Dow J. M. . Biofilm dispersal in *Xanthomonas campestris* is controlled by cell-cell signaling and is required for full virulence to plants. Proc Natl Acad Sci. 100, 10995–11000 (2003). 12960398 10.1073/pnas.1833360100 PMC196915

[b50] Wei K. . *hpaR*, a putative marR family transcriptional regulator, is positively controlled by HrpG and HrpX and involved in the pathogenesis, hypersensitive response, and extracellular protease production of *Xanthomonas campestris* pathovar campestris. J Bacteriol. 189, 2055–2062 (2007). 17158655 10.1128/JB.01331-06 PMC1855773

[b51] Chao N. X. . The *rsmA*-like gene *rsmA* (*Xcc*) of *Xanthomonas campestris* pv. *campestris* is involved in the control of various cellular processes, including pathogenesis. Mol Plant Microbe Interact. 21, 411–423 (2008). 18321187 10.1094/MPMI-21-4-0411

