# Supplementary material for: Xanthomonas campestris FabH is required for branched-chain fatty acid and DSF-family quorum sensing signal biosynthesis
Source: Sci Rep. 2016 Sep 6;6:32811. doi: 10.1038/srep32811 (PMC5011732; doi:10.1038/srep32811)
Supplement: Supplementary Information [file srep32811-s1.doc]

***Xanthomonas*** *campestris* **FabH is required for branched-chain fatty acid and DSF-family quorum sensing signal biosynthesis**

Yong-Hong Yu^1,2^, Zhe Hu^1^, Jin-Cheng Ma^1^, Hui-Juan Dong,^1^ and Hai-Hong Wang^1*^

**Supplementary Tables**

Table S1. Bacterial strains and plasmids used in this study

| **Bacterial strains** | **Relevant characteristics*^a^*** | **Source** |
| --- | --- | --- |
| ***E. coli*** |  |  |
| **DH-5α** | F^-^ *deoR* *endA1 gyrA96 hsdR17*(r_K_^-^m_K_^+^) *recA1* *relA1* *supE*44 *thi-1* Δ(*lacZYA-argF*)*U*169(φ80*lacZ*ΔM15) | Lab stock |
| **BL21(DE3)** | F^-^ *dcm* *omp T* *hsdS*(r_B_^-^m_B_^-^) *gal* (λDE3) | Lab stock |
| **S17-1** | Tp^r^ Sm^r^ *recA* *thi* *pro* *hsdR* (RP4-2 Tc::Mu Km::Tn*7*), λ*pir* | Lab stock |
| **MG1655** | Wild type | Lab stock |
| ***R. solanacearum*** | |  |
| **GMI1000** | Cm^r^, Wild-type strain | ATCC |
| **RsmH** | Cm^r^, GMI1000 Δ *fabH* | 35 |
| **RsYH1** | Cm^r^, Km^r^, GMI1000 Δ*fabH/* pSRK- *fabH_Xcc_* | This study |
| ***X. campestris* pv. *campestris*** |  |  |
| 8004 | Rif^r^, wild type | 32 |
| **8523** | Rif^r^, Tc^r^, *rpfF*::Tn5*lac* | 40 |
| **YH1** | Rif^r^, Km^r^, *Xcc* 8004 *fabH*::pZTT-1 | This study |
| **T-3** | Rif^r^, *Xcc* 8004 *fabH::EcfabH* | This study |
| **YH4** | Rif^r^, Km^r^, *Xcc* T-3/pYYH-1 | This study |
| **EcH** | Rif^r^, Gm^r^, *Xcc* 8004 Δ*fabH/* pSRK-*EcfabH* | This study |
| **Plasmids** |  |  |
| **pET-28b** | Km^r^, T7 promoter-based expression vector | Novagen |
| **pMD19** | Amp^r^, TA cloning vector | Takara |
| **pSRK-Km** | Km^r^, broad-host-range expression vector containing *lac* promoter and *lacI*^q^, *lacZ*α^+^ | 38 |
| **pSRK-Gm** | Gm^r^, broad-host-range expression vector containing *lac* promoter and *lacI*^q^, *lacZ*α^+^ | 38 |
| **pK18mobscaB** | Km^r^, *sacB*-based gene replacement vector | 39 |
| **pSRK-*EcfabH*** | Gm^r^, *EcfabH* gene cloned into plasmid pSRK-Gm | Laboratory collection |
| **pYYH-1** | Km^r^, *XccfabH* cloned into plasmid pSRK-Km | This study |
| **pYYH-2** | Km^r^, *XccfabH* cloned into plasmid pET-28b | This study |
| **pYYH-3** | Km^r^, *ΔXccfabH* inserted into pK18mobscaB between EcoRI/HindIII sites | This study |
| **pZTT-1** | Km^r^, *XccfabH* in-frame deletion fragment inserted into pK18mobscaB between EcoRI/HindIII sites | This study |
| **pZTT-2** | Amp^r^, *E.coli fabH* cloned into pMD19 | This study |
| **pZTT-3** | Km^r^, *EcfabH* gene inserted into pZTT-1 between NdeI/BamHI sites | This study |

ATCC = American Type Culture Collection; *E. coli* = *Escherichia coli*.

Table S2. Sequences of the PCR primers used

| **Primer name** | **Primer sequence (5′ to 3′)** | **Digestion sites ^a^** |
| --- | --- | --- |
| XcfabH1 EcoRI | AATTGAATTCGCCAGCGCAGCCTGCAGC | EcoRI |
| XcfabH2 | GCAGATCTAGACGGATCCCATATGGTTCCTTGGTGCAAGAGCCG | XbaI, NdeI |
| XcfabH3 | AACCATATGGGATCCGTCTAGATCTGCGCAGGCACTGCCG | XbaI, NdeI |
| XcfabH4 HindIII | AATTAAGCTTGTAGATTCGGTCACGCGTTG | HindIII |
| XcfabH NdeI | AATTCATATGAGCAAGCGGATCTACTC | NdeI |
| XcfabH HindIII | AATTAAGCTTCCCGCAATGGCAAGGCTC | HindIII |
| EcfabH NdeI | AATTCATATGTATACGAAGATTATTGGTACTGG | NdeI |
| EcfabH XbaI | TATATCTAGACGAAACGAACCAGCGCGGAG | XbaI |
| XcfabH check up | AGGTCGCCTATGTCGAACTC |  |
| XcfabH check down | CTGGCCGGGAAACACGAA |  |
| XccfabH P1−EcoRI | AATTGAATTCACCAGACCACCAGCGACC | EcoRI |
| XccfabH P2−HindIII | AATTAAGCTTCCTTGACGGCGTACTTGAACA | HindIII |

*^a^*The underlined nucleotide sequences are digestion sites of restriction endonuclease. PCR = polymerase chain reaction.

**Table S3. Fatty acid composition of total lipid extracts from *R. solanacearum* GMI1000 and mutant strain RsmH/ pYYH1 grown on BG medium***^a^*

| **Fatty acids *^b^*** | **Composition (%)** | |
| --- | --- | --- |
|  | **GMI1000** | **RsmH/pYYH1** |
| n-C_14:0_ | 5.79±0.08 | 1.26±0.14 |
| *iso*-C_15:0_ | 0.25±0.07 | 7.2±0.54 |
| *anteiso*-C_15:0_ | 0.63±0.06 | 0.72±0.11 |
| n-C_15:0_ | 0.44±0.03 | 5.5±1.59 |
| 3-OH-C_14:0_ | 21.47±1.21 | 9.38±2.05 |
| *iso*-C_16:0_ | 0 | 6.11±0.21 |
| n-C_16:1_ | 28.77±0.41 | 19.68±0.55 |
| n-C_16:0_ | 19.24±0.41 | 9.2±1.59 |
| *iso*-C_17:0_ | 0 | 13.01±0.71 |
| *anteiso*-C_17:0_ | 0 | 6.76±0.99 |
| n-C_18:2_ | 0.82±0.31 | 2.92±0.29 |
| n-C_18:1_ | 15.77±1.4 | 13.49±1.74 |
| n-C_18:0_ | 6.81±1.25 | 4.79±0.62 |
|  |  |  |
| Total UFAs | 45.36±2.13 | 36.09±2.57 |
| Total BCFAs | 0.87±0.13 | 33.78±2.56 |

*^a^*Cells were grown in BG medium for 36 h at 28°C. The total lipids were extracted and trans-esterified to obtain fatty acid methyl esters, and the products were identified by gas chromatography-mass spectrometry (GC-MS). The values are percentages of total fatty acids and are the means ± the standard deviations of three independent experiments.

***^b^***n-C_14:0_, 3-tetradecanoic; *iso*-C_15:0_, 13-methyl-tetradecanoic acid; *anteiso*-C_15:0_, 12-methyl-tetradecanoic acid; n-C_15:0_, pentadecanoic acid; 3-OH-C_14:0_, 3-hydroxytetradecanoic; *iso*-C_16:0_, 14-methyl-pentadecanoic acid; n-C_16:1_, *cis*-9-hexadecenoic acid; n-C_16:0_, hexadecanoic acid; *iso*-C_17:0_, 15-methyl-hexadecanoic acid; *anteiso*-C_17:0_, 14-methyl-hexadecanoic acid; n-C_18:2_, *cis*-11-*cis*-9-octadecenoic; n-C_18:1_, *cis*-11-octadecenoic acid; n-3-C_18:0_, octadecanoic acid. UFA indicates unsaturated fatty acid; BCFA indicates branch-chain fatty acid.

**Table S4. Fatty acid composition of total lipid extracts from *Xcc* 8004 and mutant strains grown in SXFM medium***^a^*

| **Fatty acids** *^b^* | **Composition (%)** | | | | | |
| --- | --- | --- | --- | --- | --- | --- |
|  | ***Xcc* 8004** | | ***Xcc* EcH** | | ***Xcc* T-3** | |
|  | 30°C | 15°C | 30°C | 15°C | 30°C | 15°C |
| *iso*-C_14:0_ | 0.32±0.06 | 0.59±0.07 | 0.12±0.11 | 0.25±0.06 | 0.08±0.14 | 0.21±0.06 |
| n-C_14:0_ | 1.09±0.17 | 2.04±0.22 | 2.56±0.31 | 2.31±0.45 | 2.66±0.23 | 4.11±0.41 |
| *iso*-C_15:0_ | 7.16±0.36 | 5.19±0.13 | 0 | 0 | 0 | 0 |
| *anteiso*-C_15:0_ | 20.58±0.24 | 13.53±0.45 | 0 | 0 | 0 | 0 |
| n-C_15:0_ | 3.12±0.87 | 3.01±1.02 | 0.60±0.10 | 0.2±0.1 | 0.24±0.06 | 0.20±0.02 |
| *iso*-C_16:0_ | 4.26±0.12 | 3.27±0.2 | 0 | 0 | 0 | 0 |
| n-C_16:1_ | 25.54±0.75 | 40.44±1.05 | 47.82±2.69 | 47.15±2.09 | 40.36±1.73 | 49.68±3.22 |
| n-C_16:0_ | 18.89±0.23 | 17.14±1.35 | 31.30±0.16 | 27.54±0.68 | 27.88±1.68 | 28.19±2.04 |
| *iso*-C_17:0_ | 3.27±0.06 | 2.49±0.08 | 0 | 0 | 0 | 0 |
| *anteiso*-C_17:0_ | 2.75±0.11 | 1.30±0.04 | 0 | 0 | 0 | 0 |
| n-C_17:1_ | 2.2±0.11 | 2.43±0.04 | 0.63±0.63 | 0 | 3.30±0.32 | 0 |
| n-C_18:2_ | 1.05±0.08 | 0.57±0.10 | 1.10±0.41 | 1.10±0.20 | 1.38±0.81 | 0.90±0.19 |
| n-C_18:1_ | 7.45±0.32 | 6.31±1.15 | 12.67±1.31 | 18.63±1.70 | 18.11±0.87 | 13.61±1.68 |
| n-C_18:0_ | 2.32±0.29 | 1.70±0.21 | 3.20±1.02 | 2.84±0.58 | 6.01±1.85 | 3.11±0.36 |
|  |  |  |  |  |  |  |
| Total UFAs | 36.25±1.27 | 49.75±2.34 | 62.23±5.04 | 66.88±3.99 | 63.15±3.73 | 64.19±5.09 |
| Iso-BCFAs | 15.00±0.61 | 11.54±0.47 | 0.12±0.11 | 0.25±0.06 | 0.08±0.14 | 0.21±0.06 |
| Anteiso-BCFAs | 23.34±0.35 | 14.83±0.49 | 0 | 0 | 0 | 0 |
| Total BCFAs | 38.34±0.95 | 26.37±0.96 | 0.12±0.11 | 0.25±0.06 | 0.08±0.14 | 0.21±0.06 |

*^a^*Cells were grown in BG medium for 36 h at 28°C. The total lipids were extracted and trans-esterified to obtain fatty acid methyl esters, and the products were identified by GC-MS. The values are percentages of total fatty acids and are the means ± the standard deviations of three independent experiments.

***^b^*** n-C_14:0_, 3-tetradecanoic; *iso*-C_15:0_, 13-methyl-tetradecanoic acid; *anteiso*-C_15:0_, 12-methyl-tetradecanoic acid; n-C_15:0_, pentadecanoic acid; 3-OH-C_14:0_, 3-hydroxytetradecanoic; *iso*-C_16:0_, 14-methyl-pentadecanoic acid; n-C_16:1_, *cis*-9-hexadecenoic acid; n-C_16:0_, hexadecanoic acid; *iso*-C_17:0_, 15-methyl-hexadecanoic acid; *anteiso*-C_17:0_, 14-methyl-hexadecanoic acid; n-C_18:2_, *cis*-11-*cis*-9-octadecenoic; n-C_18:1_, *cis*-11-octadecenoic acid; n-3-C_18:0_, octadecanoic acid. UFA indicates unsaturated fatty acid; BCFA indicates branch-chain fatty acid.

**Supplementary Figures**

**Figure S1**

**Fig. S1.** **Fatty acid biosynthesis catalyzed by XccFabH with mid-chain acyl-CoAs as substrates.** A. Hexanoyl-CoA as substrate. Lane 1, octanoyl-ACP (C_8:0_-ACP). Lane 2, the product of reaction with hexanoyl-CoA as substrate. Lane 3, hexanoyl-ACP (C_6:0_-ACP). B. Octanoyl-CoA as substrate. Lane 1, octanoyl-ACP (C_8:0_-ACP). Lane 2, the product of reaction with octanoyl-CoA as substrate. Lane 3, decanoyl-ACP (C_10:0_-ACP). C. Decanoyl CoA as substrate. Lane 1, decanoyl-ACP (C_10:0_-ACP). Lane 2, the product of reaction with decanoyl CoA. Lane 3, dodecanoyl-ACP (C_12:0_-ACP). D. Dodecanoyl-CoA as substrate. Lane 1, tetradecanoyl-ACP (C_14:0_-ACP). Lane 2, the product of reaction with dodecanoyl-CoA. Lane 3, dodecanoyl-ACP (C_12:0_-ACP).

**Figure S2**

**Fig. S2. A. Strategy for isolation of the *Xcc* *fabH* deletion mutant strain. B. Genetic organization of the *fabH* region in *fabH* merodiploid strain *Xcc* YH1. C. Polymerase chain reaction (PCR) analyses of genomic DNA from the strain depicted in (B).** Abbreviations: CH, chromosome; Up, the upstream fragment of *fabH*; Dn, the downstream fragment of *fabH*; p1, primer XcfabH1 EcoRI listed in Table S2; p2, primer XcfabH4 HindIII listed in Table S2. E. coli = *Escherichia coli*.

**Figure S3**

**Fig. S3. Strategy for isolation of the *Xcc* *fabH* insertion mutant strain.** Abbreviations: CH, chromosome; E. coli, *Escherichia coli*

**Figure S4**

**Fig. S4.** **A. Strategy for isolation of the *Xcc* T-3 (*XccfabH*::*EcfabH*) mutant strain that *Xcc* *fabH* was replaced in fame with *Escherichia coli* (*E. coli*) *fabH*. B. Genetic organization of the *fabH* region in strain *Xcc* 8004 (a) or *Xcc* T-3 (b). C. Polymerase chain reaction (PCR) analyses of genomic DNA from the strains depicted in (B).** Abbreviations: CH, chromosome; Up, the upstream fragment of *XccfabH*; Dn, the downstream fragment of *XccfabH*; p3, primer EcfabH NdeI listed in Table S2; p4, primer EcfabH XbaI listed in Table S2.

**Figure S5**

**Fig. S5.** **A. Strategy for isolation of *Xcc* EcH (Δ*XccfabH*/pSRK-*EcfabH*) mutant, in which *Escherichia coli (E. coli) fabH* was expressed from plasmid pSRK-Gm and the *Xcc fabH* gene was deleted from its genome. B. Genetic organization of the *fabH* region in strain *Xcc* 8004 (a) or *Xcc* EcH (b). C. Polymerase chain reaction (PCR) analyses of genomic DNA from the strains depicted in (B).** Abbreviations: CH, chromosome; Up, the upstream fragment of *XccfabH*; Dn, the downstream fragment of *XccfabH*; p1, primer XcfabH1 EcoRI listed in Table S2; p2, primer XcfabH4 HindIII listed in Table S2.

**Figure 6S**

**Fig. S6. A. Growth of *Xcc* strains in Chinese cabbage juice.** Chinese cabbage juice was prepared as described in “Material and Methods”. The bacterial growth was recorded as turbidity using an automatic growth analyzer (BioScreen, Labsystems) at 600 nm. **B. In the plants, bacterial growth of *Xcc* strains after inoculation of Chinese cabbage stem discs was determined.** Inoculation of Chinese cabbage stem discs with Xcc strains was described in “Material and Methods”. Cell numbers were determined by the plate method. Squares indicate *Xcc* 8004; triangles indicate *Xcc* EcH; diamonds indicate *Xcc* T-3; and circles indicate *Xcc* 8523.
